# CD146 is a Novel ANGPTL2 Receptor that Promotes Obesity by Manipulating Lipid Metabolism and Energy Expenditure

**DOI:** 10.1002/advs.202004032

**Published:** 2021-01-27

**Authors:** Zhenzhen Wu, Jingyu Liu, Gang Chen, Junfeng Du, Huiyun Cai, Xuehui Chen, Gaoqi Ye, Yongting Luo, Yiyi Luo, Liwen Zhang, Hongxia Duan, Zheng Liu, Sai Yang, Hongwei Sun, Yan Cui, Lei Sun, Hongjie Zhang, Guizhi Shi, Taotao Wei, Pingsheng Liu, Xiyun Yan, Jing Feng, Pengcheng Bu

**Affiliations:** ^1^ Key Laboratory of RNA Biology Key Laboratory of Protein and Peptide Pharmaceuticals Institute of Biophysics Chinese Academy of Sciences Beijing 100101 China; ^2^ College of Life Sciences University of Chinese Academy of Sciences Beijing 100049 China; ^3^ Key Laboratory of Protein and Peptide Pharmaceuticals Institute of Biophysics Chinese Academy of Sciences Beijing 100101 China; ^4^ Medical Department of General Surgery Chinese PLA General Hospital Beijing 100853 China; ^5^ Department of General Surgery 7th Medical Center Chinese PLA General Hospital Beijing 100700 China; ^6^ The Second School of Clinical Medicine Southern Medical University Guangdong 510515 China; ^7^ Savaid Medical School University of Chinese Academy of Sciences Beijing 100049 China; ^8^ Laboratory Animal Research Center Institute of Biophysics Chinese Academy of Sciences Beijing 100101 China; ^9^ Department of Hepatobiliary Surgery Beijing 306 Hospital Beijing 100101 China; ^10^ Center for Biological Imaging Institute of Biophysics Chinese Academy of Sciences Beijing 100101 China; ^11^ The Core Facilities Institute of Biophysics Chinese Academy of Sciences Beijing 100101 China; ^12^ National Laboratory of Biomacromolecules Institute of Biophysics Chinese Academy of Sciences Beijing 100101 China; ^13^ Center for Excellence in Biomacromolecules Chinese Academy of Sciences Beijing 100101 China

**Keywords:** ANGPTL2, brown adipocyte, CD146, energy expenditure, obesity, white adipocyte

## Abstract

Obesity and its related complications pose an increasing threat to human health; however, targetable obesity‐related membrane receptors are not yet elucidated. Here, the membrane receptor CD146 is demonstrated to play an essential role in obesity. In particular, CD146 acts as a new adipose receptor for angiopoietin‐like protein 2 (ANGPTL2), which is thought to act on endothelial cells to activate adipose inflammation. ANGPTL2 binds to CD146 to activate cAMP response element‐binding protein (CREB), which then upregulates CD146 during adipogenesis and adipose inflammation. CD146 is present in preadipocytes and mature adipocytes, where it is mediated by its ligands ANGPTL2 and galectin‐1. In preadipocytes, CD146 ablation suppresses adipogenesis, whereas the loss of CD146 in mature adipocytes suppresses lipid accumulation and enhances energy expenditure. Moreover, anti‐CD146 antibodies inhibit obesity by disrupting the interactions between CD146 and its ligands. Together, these findings demonstrate that ANGPTL2 directly affects adipocytes via CD146 to promote obesity, suggesting that CD146 can be a potential target for treating obesity.

## Graphical abstract

The membrane receptor CD146 is a novel ANGPTL2 receptor that promotes obesity and insulin resistance. By interacting with ANGPTL2 and galectin‐1, CD146 enhances adipogenesis and lipogenesis while suppressing FAO and BAT thermogenesis. Furthermore, the disruption of the interaction between CD146 and its ligands by knocking out CD146 or using anti‐CD146 antibodies may prevent obesity and its related complications.

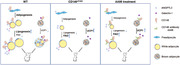

## 1 Introduction

Obesity has become one of the major global health challenges of the 21st century, with an increasing global incidence expected to lead to higher rates of morbidity and mortality due to obesity‐related complications, such as chronic inflammatory diseases like insulin resistance and cardiovascular diseases.^[^ ^1^, ^2^ ^]^ Obesity is mainly caused by adipose tissue expansion due to adipocyte hyperplasia and hypertrophy.

Adipocyte hyperplasia is the result of preadipocyte differentiation via adipogenesis, which is regulated by transcription factors such as CREB, CCAAT/enhancer binding protein (C/EBP) *α*, *β*, *δ*, and peroxisome proliferator‐activated receptor *γ* (PPAR*γ*). After preadipocyte differentiation has been initiated, elevated intracellular cAMP levels activate protein kinase A (PKA), which phosphorylates CREB and upregulates C/EBP*β*, C/EBP*α*, and PPAR*γ* expression.^[^ ^3^, ^4^, ^5^ ^]^ The transcription factors KLF5, Zfp423, and COUP‐TFII also play key roles in adipogenesis.^[^ ^6^, ^7^, ^8^ ^]^

Conversely, adipocyte hypertrophy is caused by the over‐accumulation of lipids due to unbalanced lipid metabolism and low energy expenditure, such as high rates of lipid uptake and lipogenesis as well as low rates of lipolysis, fatty acid oxidation, and brown adipose tissue (BAT) thermogenesis.^[^ ^9^, ^10^ ^]^ Lipid accumulation in white adipose tissue (WAT) is mainly regulated by hormones and adipocytokines, including insulin, adiponectin, leptin, and ANGPTL2.^[^ ^11^, ^12^ ^]^ Although studies of intracellular transcription factors and extracellular cytokines have yielded major advances in our understanding of adipogenesis and lipid accumulation, the receptors linking extracellular adipocytokines to intracellular transcription factors remain largely unknown.

ANGPTL2 is a mediator protein that is critically involved in obesity‐related chronic inflammation and its overexpression has been reported to result in vascular inflammation, increased macrophage infiltration, and increased inflammatory cytokine (TNF‐*α*, IL‐6, and IL‐1*β*) expression in adipose tissue.^[^ ^13^, ^14^ ^]^ In addition, ANGPTL2 was found to be upregulated in adipose tissue during obesity, insulin resistance, and obesity‐related inflammation in both mice and humans,^[^ ^13^, ^15^, ^16^ ^]^ while ANGPTL2 deficiency was shown to ameliorate high‐fat diet (HFD)‐induced obesity and adipose tissue inflammation.^[^ ^13^ ^]^ Although ANGPTL2 clearly plays a key role in obesity, it remains unclear how ANGPTL2 links adipose inflammation to obesity.

CD146 is a membrane receptor that was originally found to be highly expressed in metastatic lesions and advanced primary tumors;^[^ ^17^, ^18^ ^]^ however, experimental evidence also suggests that CD146 is a multifunctional cell adhesion molecule with crucial roles in angiogenesis and inflammatory diseases.^[^ ^19^, ^20^, ^21^, ^22^, ^23^ ^]^ Recently, we demonstrated that CD146 interacts with CD36, a receptor responsible for ox‐LDL uptake, to promote foam cell formation and retention during atherosclerosis;^[^ ^24^ ^]^ thus, CD146 may affect obesity development and obesity‐related chronic inflammation.

In this study, we investigated patterns of CD146 expression in adipocytes and WAT from obese mice and humans. Having identified adipocyte CD146 as a novel ANGPTL2 receptor, we investigated its regulatory mechanisms and effects on obesity in vitro and in vivo. Together, our findings demonstrate that cooperation between CD146 and its ligands plays a critical role in obesity.

## 2 Results

### 2.1 CD146 Expression Is Elevated in Fat Tissue During Obesity

**Figure 1:**
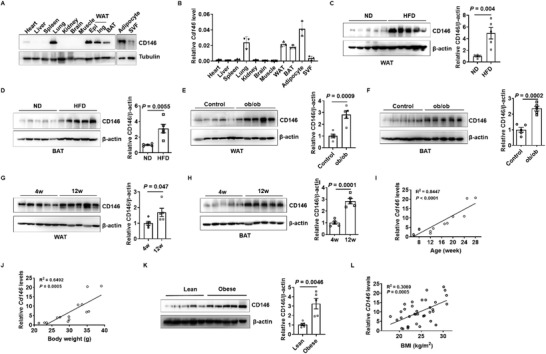
CD146 expression is upregulated in adipose tissues from obese mice and humans. Western blot A) and RT‐qPCR B) showing CD146 levels in various mouse tissues. SVF, stromal vascular fraction. Western blot showing CD146 expression in white adipose tissue (WAT) C) and brown adipose tissue (BAT) D) from mice fed a normal diet (ND) or high‐fat diet (HFD) (*n* = 5 per group). Western blot showing CD146 expression in WAT E) and BAT F) from 12 week old (12w) ob/ob or control mice (*n* = 5 per group). Western blot showing CD146 expression in WAT G) and BAT H) from 4 week old (4w) or 12w ob/ob mice (*n* = 5 per group). I) Correlation between CD146 levels in WAT and the age of mice (*n* = 14). J) Correlation between CD146 levels in WAT and the body weight of mice (*n* = 14). K) Western blot showing CD146 expression in WAT from lean and obese humans. L) Correlation between CD146 levels in human WAT and body mass index (*n* = 36). Data represent the mean ± SD in (B) and the mean ± SEM in (C)–(H) and (K). *P* values were determined using Student's *t*‐tests (C–H,K).

Preliminary analysis of the BioGPS database (http://biogps.org/#goto=welcome) indicated that CD146 is highly expressed in the adipose tissue of both mice and humans (Figure S1, Supporting Information); therefore, we systematically analyzed CD146 expression in various mouse tissues, including the heart, liver, spleen, lung, kidney, brain, muscle, WAT from epididymal (Epi) and inguinal (Ing) fat pads, and BAT. CD146 was highly expressed in both WAT and BAT (**Figure** **1**A,B), which contain the adipocyte fraction and stromal vascular fraction (SVF). Western blot and RT‐qPCR analyses further indicated that CD146 was strongly expressed in adipocytes, but only weakly expressed in the SVF (Figure 1A,B), confirming that CD146 is highly expressed in adipose tissue and particularly in adipocytes.

To further elucidate the correlation between obesity and CD146 expression, we examined CD146 expression in adipose tissue from high‐fat diet‐induced and leptin deletion‐induced obese mice (ob/ob mice). We observed that CD146 expression was significantly upregulated in both the WAT and BAT of obese mice compared to the normal controls (Figure 1C–F) and was 63% and 80% higher in WAT and BAT from 12 week old (12w) ob/ob mice compared to 4 week old (4w) ob/ob mice (Figure 1G,H). In addition, CD146 upregulation mainly occurred in adipocytes, but not the SVF (Figure S2, Supporting Information). The integrated analysis of CD146 expression and body weight in wild‐type (WT) mice at various ages further confirmed that CD146 expression correlated positively with age‐induced obesity (Figure 1I,J). To determine the clinical relevance of CD146 expression in obesity, we analyzed CD146 expression in visceral fat from obese and lean human subjects, finding that CD146 expression was significantly higher in the visceral fat of obese individuals and correlated positively with body mass index (BMI; Figure 1K,L). Together, these findings suggest that CD146 is upregulated in the WAT of obese mice and humans.

### 2.2 CD146 Knockout Protects Mice from Obesity

**Figure 2:**
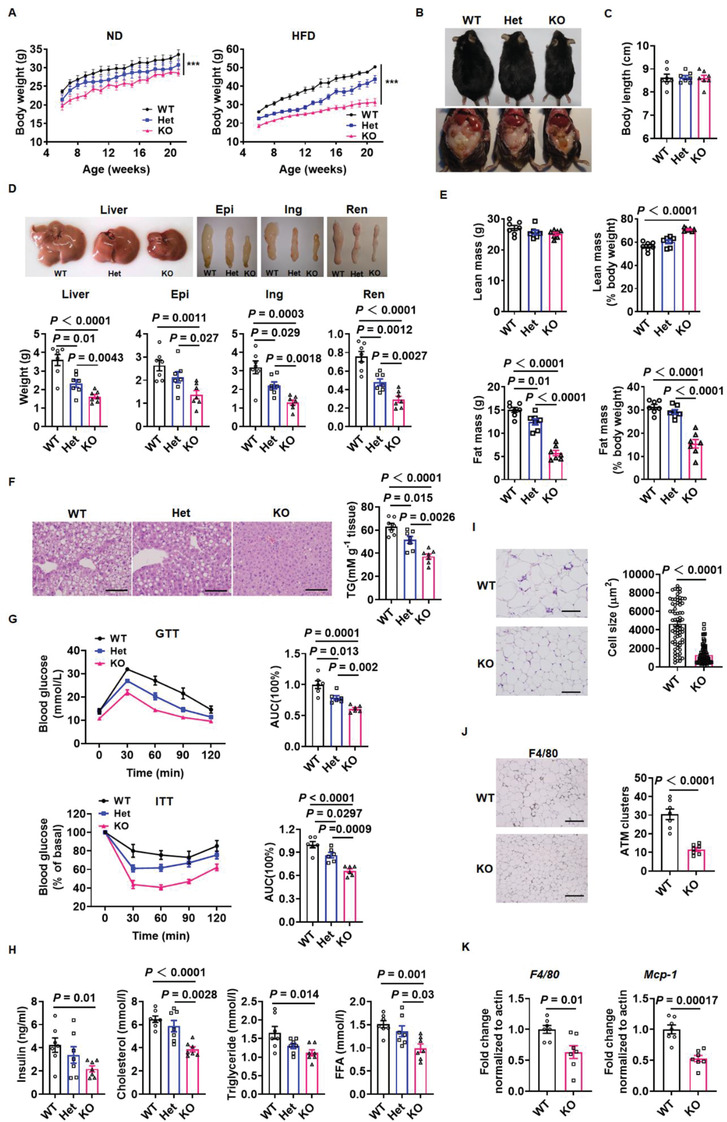
CD146 knockout protects mice from obesity. A) Body weight of wild‐type (WT), CD146 heterozygous knockout (Het), and knockout (KO) mice fed a normal diet (ND) or high‐fat diet (HFD) (*n* = 7 per group). B) Representative images of WT, Het, and KO mice fed an HFD. C) Body length of WT, Het, and KO mice fed an HFD (*n* = 7 per group). D) Representative images and weight of liver and fat pads (epididymal, inguinal, and renal) from WT, Het, and KO mice fed an HFD (*n* = 7 per group). E) Lean and fat mass of WT, Het, and KO mice fed an HFD, determined by micro‐CT (*n* = 7 per group). F) Representative H&E images (left) and triglyceride (TG) content (right) of livers from WT, Het, and KO mice fed an HFD (*n* = 7 per group). Scale bar, 100 µm. G) Glucose tolerance test (GTT) and insulin tolerance test (ITT) in WT, Het, and KO mice fed an HFD (*n* = 6 per group). H) Serum insulin, cholesterol, triglyceride, and free fatty acid levels in fasting WT, Het, and KO mice (*n* = 7 per group). I) Representative H&E images and adipocyte cell size measurements of epididymal fat pads from WT and KO mice fed an HFD. Scale bar, 100 µm. J) Representative F4/80 staining images and quantification of F4/80 positive adipose tissue macrophage (ATM) clusters in epididymal fat pads from WT and KO mice fed an HFD. Scale bar, 200 µm. K) RT‐qPCR analysis of *F4/80* and *MCP‐1* levels in epididymal fat pads from WT and KO mice fed an HFD. Data represent the mean ± SEM. *P* values were determined using one‐way ANOVA in (A)–(H) and Student's *t*‐tests in (I)–(K).

To better understand how elevated CD146 levels are linked to obesity, we generated CD146 knockout (KO) and heterozygous knockout (Het) mice, with wildtype (WT) littermates as controls (Figure S3, Supporting Information). When the mice were fed a normal diet (ND), the body weight and fat mass of CD146 KO mice were lower than those of the WT mice (**Figure** **2**A; Figure S4B, Supporting Information), whereas the lean mass and weight of most other tissues (heart, liver, spleen, lung, and kidney) from CD146 KO mice did not change significantly (Figure S4, Supporting Information). When fed an HFD, CD146 KO mice exhibited significantly lower body weight (36%) and were leaner than WT mice (Figure 2A,B), while their body lengths were similar (Figure 2C). The liver and fat pads are major lipid‐containing tissues, among which the epididymal (Epi), inguinal (Ing), and renal (Ren) fat pad weights were significantly lower in CD146 KO mice than in WT mice (Figure 2D), suggesting that the lower body weight of KO mice is mainly due to reduced liver and WAT weight. To confirm the changes in fat mass between WT and KO mice, we analyzed body composition using micro‐CT, finding that fat mass was significantly lower in CD146 KO mice, yet their lean body mass was higher (Figure 2E). Taken together, these results show that the absence of CD146 protects mice from HFD‐induced obesity, primarily by reducing fat mass.

### 2.3 CD146 Knockout Reduces Insulin Resistance and Adipose Inflammation

Ectopic lipid accumulation results in lipotoxic metabolic stress, which promotes metabolic dysfunction in organs such as the liver.^[^ ^25^ ^]^ To investigate whether CD146 enhances lipid accumulation in the liver, we performed H&E staining in livers from WT, CD146 Het, and CD146 KO mice. The livers of CD146 KO mice contained fewer lipid‐containing vacuoles than those of WT mice (Figure 2F, left). We quantified this further by measuring the triglyceride (TG) content of the livers (Figure 2F, right), finding that CD146 KO livers contained fewer TGs than WT livers.

Obesity is associated with many health problems, including insulin resistance and chronic inflammation.^[^ ^26^ ^]^ Insulin resistance leads to a compensatory increase in plasma insulin levels and decreased glucose uptake.^[^ ^27^ ^]^ When fed an HFD, the WT mice exhibited lower insulin sensitivity and glucose tolerance, whereas CD146 knockout significantly improved insulin sensitivity and glucose tolerance (Figure 2G). In addition, fasting insulin levels were lower in CD146 KO mice fed an HFD (Figure 2H), while CD146 KO mice fed an HFD displayed significantly lower serum cholesterol (CHO), TG, and free fatty acid (FFA) levels during fasting (Figure 2H).

Adipose tissue enlargement is associated with a prominent inflammatory response in the visceral compartment and infiltrating macrophages in WAT are key mediators of this inflammatory process.^[^ ^28^ ^]^ Significantly smaller adipocytes and significantly fewer F4/80 positive macrophages were observed in the epididymal fat of CD146 KO mice fed an HFD compared to WT mice (Figure 2I,J). Moreover, RT‐qPCR revealed that the expression of macrophage marker genes (*F4/80* and monocyte chemoattractant protein‐1 (*Mcp‐1*)) were markedly lower in the epididymal fat of CD146 KO mice (Figure 2K). Overall, these findings suggest that CD146 mediates the detrimental effects of HFD‐induced obesity, such as insulin resistance, fatty liver, and inflammation.

### 2.4 CD146 Knockout Increases Energy Expenditure

Obesity mainly occurs due to an imbalance in energy homeostasis, high energy intake, and low energy expenditure.^[^ ^29^ ^]^ Although energy intake is normally reflected by food intake, we found that CD146 KO mice consumed more food than WT mice on either the ND or HFD (Figure S5A, Supporting Information), seeming to contradict the observation that CD146 KO mice were leaner than WT mice. Therefore, we investigated the energy expenditure of WT and CD146 KO mice, finding that both had similar physical activity levels on the ND and HFD (Figure S5B, Supporting Information). However, CD146 KO mice had approximately 21% higher oxygen consumption (VO_2_), 12% higher carbon dioxide production (VCO_2_), 7% lower respiratory quotient (VCO_2_/VO_2_), and 19% higher energy expenditure than WT mice on the ND (Figure S5C–F, left, Supporting Information). Moreover, CD146 KO mice fed an HFD exhibited a 21% increase in VO_2_, a 13% increase in VCO_2_, 7% lower VCO_2_/VO_2_, and a 20% increase in total energy expenditure relative to WT mice (Figure S5C–F, right, Supporting Information), indicating that CD146 KO mice expend more energy.

As an energy expenditure tissue, BAT can dissipate energy in the form of heat.^[^ ^30^ ^]^ To determine whether CD146 affected BAT function, we isolated interscapular BAT from WT and CD146 KO mice. The weight and mass of BAT did not significantly differ between CD146 KO and WT mice when fed an ND (Figure S5H,I, left, Supporting Information); however, BAT from CD146 KO mice had a more intense color than that of WT mice (Figure S5G, left, top, Supporting Information), indicating a reduced lipid content. H&E staining further confirmed that CD146 KO BAT contained fewer multilocular lipid droplets than WT BAT (Figure S5G, left, bottom, Supporting Information); furthermore, BAT weight and lipid content significantly increased in WT mice compared to CD146 KO mice when fed an HFD, whereas BAT mass was similar (Figure S5G–I, right, Supporting Information).

Mitochondrial biogenesis is positively related to BAT thermogenesis;^[^ ^31^ ^]^ therefore, we examined the mitochondrial density of BAT via transmission electron microscopy (TEM). Increased mitochondrial volume density and cristae number were observed in CD146 KO BAT (Figure S5J, Supporting Information), while quantitative analysis revealed that the ratio of mitochondrial to cytoplasmic areas also increased in CD146 KO BAT (Figure S5K, Supporting Information). UCP1 is a BAT‐specific protein that is essential for BAT function.^[^ ^32^ ^]^ Consistent with the TEM results, we found that UCP1 expression was significantly higher in CD146 KO than WT BAT (Figure S5L, Supporting Information), alongside the expression of genes related to BAT thermogenesis and fatty acid oxidation (FAO; Figure S5M, Supporting Information). To confirm the respiratory function of BAT mitochondria isolated from WT or KO mice, we measured their oxygen consumption rate (OCR). Although substrate addition induced respiration in both WT and KO mitochondria, those from CD146 KO BAT exhibited increased substrate‐induced respiration (Figure S5N, Supporting Information). Furthermore, inhibiting UCP1‐dependent respiration using guanosine diphosphate (GDP) demonstrated that UCP1‐dependent respiration is increased in CD146 KO BAT mitochondria (Figure S5O, Supporting Information). Taken together, these findings suggest that CD146 deficiency enhances BAT function by increasing UCP1‐dependent mitochondrial respiration.

### 2.5 Adipose Tissue‐Specific CD146 Knockout Inhibits HFD‐Induced Obesity

**Figure 3:**
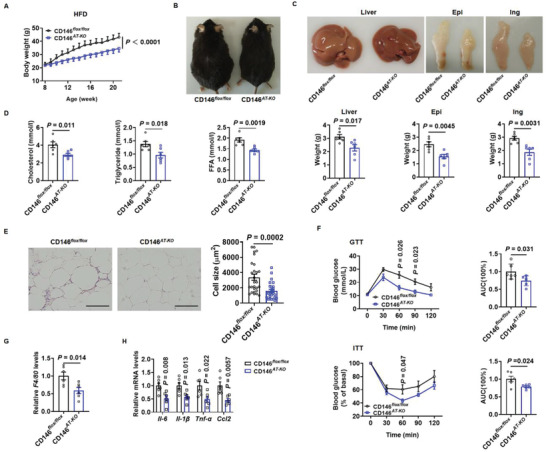
Adipocyte‐specific CD146 knockout (CD146^*AT‐KO*^) protects mice from obesity. A) Body weight of CD146^*AT‐KO*^ and CD146^*flox/flox*^ control mice fed an HFD (*n* = 6 per group). B) Representative images of CD146^*AT‐KO*^ and CD146^*flox/flox*^ control mice fed an HFD. C) Representative images and weight of liver and fat pads (epididymal and inguinal) from CD146^*AT‐KO*^ and CD146^*flox/flox*^ control mice fed an HFD (*n* = 6 per group). D) Serum cholesterol, triglyceride, and FFA levels in fasting CD146^*AT‐KO*^ and CD146^*flox/flox*^ control mice. E) Representative H&E images and adipocyte cell size measurements of epididymal fat pads from CD146^*AT‐KO*^ and CD146^*flox/flox*^ control mice fed an HFD. Scale bar, 50 µm. F) GTT and ITT measured in CD146^*AT‐KO*^ and CD146^*flox/flox*^ control mice fed an HFD (*n* = 6 per group). G) RT‐qPCR analysis of *F4/80* expression in epididymal fat pads from CD146^*flox/flox*^ and CD146^*AT‐KO*^ mice fed an HFD (*n* = 6 per group). H) RT‐qPCR analysis of the expression of the inflammatory factors *Il‐6*, *Il‐1β*, *Tnf‐α*, and *Ccl2* in epididymal fat pads from CD146^*flox/flox*^ and CD146^*AT‐KO*^ mice fed an HFD (*n* = 6 per group). Data represented the mean ± SEM. *P* values were determined using Student's *t*‐tests.

To assess the role of adipocyte CD146 in the regulation of obesity and adipose inflammation, we generated adipose‐specific CD146‐knockout (CD146^*AT‐KO*^) mice by crossing CD146^*flox/flox*^ mice with AdipoQ‐derived Cre mice (Figure S6A–C, Supporting Information). When fed an ND, body weight and fat mass were lower in CD146^*AT‐KO*^ mice than in the control CD146^*flox/flox*^ littermates, whereas lean mass did not change significantly (Figure S7A,B, Supporting Information). Conversely, when fed an HFD the CD146^*AT‐KO*^ mice gained less weight and were leaner than the control CD146^*flox/flox*^ littermates (**Figure** **3**A,B). In addition, liver and fat pad weight were lower in CD146^*AT‐KO*^ mice than in the CD146^*flox/flox*^ controls (Figure 3C), while the CD146^*AT‐KO*^ mice had smaller adipocytes and lower serum CHO, TG, and FFA levels than the CD146^*flox/flox*^ control mice (Figure 3D,E). Moreover, glucose tolerance test (GTT) and insulin tolerance test (ITT) showed that CD146 KO in adipocytes significantly improved insulin sensitivity and glucose tolerance (Figure 3F) but decreased the expression of the macrophage marker gene *F4/80* and chronic inflammation‐related genes in the visceral adipose tissue of CD146^*AT‐KO*^ mice (Figure 3G,H). Taken together, these data indicate that adipose‐specific CD146 KO prevents HFD‐induced obesity and lipid metabolism disorders, as well as significantly reducing obesity‐related insulin resistance and chronic inflammation.

### 2.6 Adipose Tissue‐Specific CD146 Knockout Enhances Energy Expenditure and BAT Function

**Figure 4:**
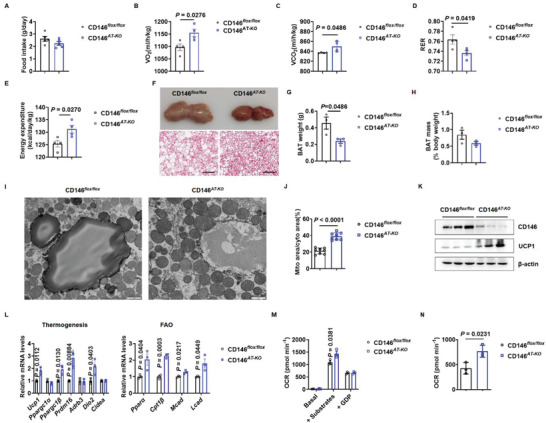
Adipocyte‐specific CD146 knockout (CD146^*AT‐KO*^) enhances energy expenditure and BAT function. A) Food intake of CD146^*AT‐KO*^ and CD146^*flox/flox*^ control mice fed an HFD measured daily (*n* = 5 per group). Oxygen consumption B), carbon dioxide production C), respiratory quotient D), and energy expenditure E) of CD146^*AT‐KO*^ and CD146^*flox/flox*^ control mice fed an HFD (*n* = 4 per group). F) Representative images (top) and H&E staining (bottom) of BAT from CD146^*AT‐KO*^ and CD146^*flox/flox*^ control mice fed an HFD. Scale bar, 200 µm. BAT weight G) and mass H) of CD146^*AT‐KO*^ and CD146^*flox/flox*^ control mice fed an HFD (*n* = 3 per group). I) Representative images of BAT mitochondria from CD146^*AT‐KO*^ and CD146^*flox/flox*^ control mice fed an HFD. Scale bar, 1 µm. J) Ratio of mitochondria to cytosol area in CD146^*AT‐KO*^ and CD146^*flox/flox*^ control mice fed an HFD. K) Western blot showing UCP1 expression in BAT from CD146^*AT‐KO*^ and CD146^*flox/flox*^ control mice fed with HFD. L) RT‐qPCR showing the expression of thermogenesis and fatty acid oxidation (FAO)‐related genes in BAT from CD146^*AT‐KO*^ and CD146^*flox/flox*^ control mice fed an HFD (*n* = 3 per group). M) Oxygen consumption rate (OCR) in mitochondria from CD146^*AT‐KO*^ and CD146^*flox/flox*^ control BAT. N) UCP1‐dependent respiration calculated from (M). Data represent the mean ± SEM. *P* values were determined using Student's *t*‐tests.

To investigate the role of adipocyte CD146 in energy homeostasis, we examined the food intake and energy expenditure of CD146^*AT‐KO*^ mice fed an ND or HFD. Although CD146^*AT‐KO*^ mice consumed similar amounts of food to the CD146^*flox/flox*^ control mice (**Figure** **4**A; Figure S7C, Supporting Information), the CD146^*AT‐KO*^ mice had higher VO_2_ and VCO_2_ rates, higher energy expenditure, and a lower respiratory quotient than the CD146^*flox/flox*^ control mice (Figure 4B–E; Figure S7D–G, Supporting Information), suggesting that CD146^*AT‐KO*^ mice expend more energy.

To determine whether adipocyte CD146 expression affects BAT function, we isolated BAT from CD146^*AT‐KO*^ and CD146^*flox/flox*^ mice fed an ND or HFD. The BAT from CD146^*AT‐KO*^ mice had a more intense color and contained fewer multilocular lipid droplets than that of CD146^*flox/flox*^ mice (Figure 4F; Figure S7H, Supporting Information). When fed an ND, BAT weight and mass were similar in CD146^*flox/flox*^ and CD146^*AT‐KO*^ mice (Figure S7I,J, Supporting Information); however, when the mice were fed an HFD the CD146^*AT‐KO*^ mice displayed a lower BAT weight than the CD146^*flox/flox*^ mice (Figure 4G), but BAT mass was similar (Figure 4H). In addition, the number of mitochondria and the ratio of mitochondrial to cytoplasmic area increased in BAT from CD146^*AT‐KO*^ mice (Figure 4I,J; Figure S7K,L, Supporting Information), as well as the expression of UCP1 and genes related to BAT function (Figure 4K,L; Figure S7M,N, Supporting Information). Expectedly, substrate‐induced and UCP1‐dependent respiration were also enhanced in BAT mitochondria from CD146^*AT‐KO*^ mice (Figure 4M,N; Figure S7O,P, Supporting Information). Thus, adipocyte‐specific CD146 knockout enhances BAT thermogenesis.

### 2.7 CD146 Promotes Adipogenesis in Preadipocytes

**Figure 5:**
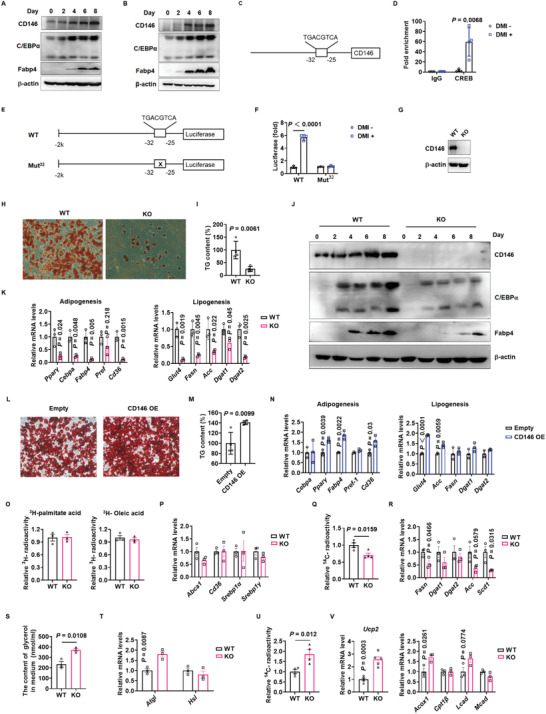
CD146 promotes adipogenesis and lipid accumulation. Western blot showing CD146, C/EBP*α*, and Fabp4 expression during the differentiation of stromal vascular fractions (SVF) A) and 3T3‐L1 cells B). C) Schematic diagram illustrating a putative CREB binding site in the CD146 promoter. D) ChIP‐qPCR analysis showing CREB binding to the CD146 promoter during DMI‐induced 3T3‐L1 differentiation. E) Schematic diagram of luciferase reporter constructs, wherein luciferase is driven by the CD146 promoter with a WT or deleted putative CREB binding site. F) Dual luciferase reporter assay confirming that CREB binds to the CD146 promoter during DMI‐induced 3T3‐L1 differentiation. CD146 expression G), oil‐red‐O staining H) and TG content I) of adipocytes differentiated from WT and CD146 KO SVF. J) Western blot showing CD146, C/EBP*α*, and Fabp4 expression during adipocyte differentiation from SVF. K) RT‐qPCR showing the expression of adipogenesis and lipogenesis‐related genes in adipocytes differentiated from SVF. L) Oil‐red‐O staining of adipocytes differentiated from 3T3‐L1 cells with ectopic CD146 expression. M) Triglyceride content in adipocytes differentiated from 3T3‐L1 cells with ectopic CD146 expression. N) RT‐qPCR analysis of adipogenesis and lipogenesis‐related gene expression in adipocytes differentiated from 3T3‐L1 cells. O) Lipid uptake measured in WT and CD146 KO adipocytes (*n* = 3 per group). P) RT‐qPCR analysis of lipid uptake‐related gene expression in WT and CD146 KO adipocytes (*n* = 3 per group). Q) ^14^C radioactivity indicating lipogenesis rate in WT and CD146 KO adipocytes (*n* = 4 per group). R) RT‐qPCR analysis of lipogenesis‐related gene expression in WT and CD146 KO adipocytes (*n* = 3 per group). S) Glycerol release measurements indicating lipolysis rate in WT and CD146 KO adipocytes (*n* = 3 per group). T) RT‐qPCR analysis of lipolysis‐related gene upregulation in WT and CD146 KO adipocytes (*n* = 3 per group). U) ^14^C radioactivity indicating fatty acid oxidation rate in WT and CD146 KO adipocytes (*n* = 4 per group). V) RT‐qPCR analysis of fatty acid oxidation‐related gene expression in WT and CD146 KO adipocytes (*Ucp2*, *n* = 5 per group; *other genes, n* = 3 per group). Data represent the mean ± SD in (A)–(N) and mean ± SEM in (O)–(V). *P* values were determined using Student's *t*‐tests.

Adipogenesis is a significant cause of obesity;^[^ ^33^ ^]^ therefore, we investigated whether CD146 contributes toward adipogenesis by examining CD146 expression in the SVF of WAT, which contains preadipocytes that differentiate into adipocytes via adipogenesis. SVF differentiation was induced using an insulin/dexamethasone/IBMX differentiation cocktail (DMI). During SVF differentiation, the expression of adipogenic genes such as C/EBP*α* and Fabp4 was gradually upregulated and CD146 expression gradually increased (**Figure** **5**A). To confirm this observation, we induced 3T3‐L1 cell differentiation using DMI, finding that CD146 and adipogenic genes were also upregulated during adipogenesis in 3T3‐L1 cells (Figure 5B).

To identify the mechanism of CD146 upregulation during DMI‐induced adipogenesis, we analyzed the CD146 promoter region using the MSigDB and JASPAR databases, both of which indicated a putative CREB binding site at −32 to −25 of the CD146 promoter (Figure 5C). Since CREB is one of the most important transcriptional activators of adipogenesis,^[^ ^34^ ^]^ we tested whether CREB bound to this putative site to upregulate CD146 during DMI‐induced adipogenesis. ChIP‐qPCR revealed that CREB was significantly enriched at the CD146 promoter in DMI‐induced 3T3‐L1 cells compared to noninduced 3T3‐L1 cells (Figure 5D), consistent with our observation that CD146 was upregulated during DMI‐induced adipogenesis. Furthermore, luciferase reporter assays confirmed that the wild‐type CD146 promoter efficiently activated firefly luciferase expression during DMI treatment, whereas luciferase was not activated following binding site mutation or no DMI treatment (Figure 5E,F). Collectively, these results demonstrate that CREB directly binds to the CD146 promoter and upregulates its expression during adipogenesis.

To investigate whether CD146 is required for adipogenesis, we induced adipocyte differentiation in SVF isolated from WT or CD146 KO mice and performed oil‐red‐O staining to evaluate lipid droplet content and western blotting to determine adipogenic gene expression 8 days after DMI induction. WT SVF displayed efficient adipocyte differentiation characterized by high lipid droplet content and adipogenesis marker expression, whereas CD146 KO SVF exhibited significantly reduced lipid droplet accumulation and adipogenesis marker expression (Figure 5G–K). In addition, we ectopically expressed CD146 in 3T3‐L1 cells (CD146 OE) and induced their differentiation into adipocytes. The CD146 OE cells exhibited a higher lipid density than the control cells (Figure 5L), higher TG levels (Figure 5M), and the increased expression of various adipogenesis‐ and lipogenesis‐related genes (Figure 5N), collectively demonstrating that CD146 is essential for adipocyte differentiation.

### 2.8 CD146 Enhances Lipid Accumulation in Mature Adipocytes

To investigate the effect of CD146 on lipid accumulation in mature adipocytes, we systematically evaluated lipid uptake, lipogenesis, lipolysis, and fatty acid oxidation in mature adipocytes isolated from WT or CD146 KO mice. The adipocytes were incubated with [^3^H]‐labeled oleic acid or palmitic acid for 5 min and lipid uptake was evaluated by measuring cellular radioactivity, while the expression of lipid uptake‐related genes was determined using RT‐qPCR. No differences in radioactivity or gene expression were observed between the WT and CD146 KO adipocytes (Figure 5O,P), indicating that CD146 KO does not affect fatty acid uptake; therefore, we analyzed lipogenesis and lipolysis in the isolated adipocytes. CD146 KO suppressed lipogenesis, as indicated by reduced nascent lipid levels and the downregulation of lipogenesis‐related genes (Figure 5Q,R). Conversely, CD146 KO enhanced lipolysis, as indicated by increased glycerol release and the upregulation of lipolysis‐related genes (Figure 5S,T). To evaluate the effect of CD146 KO on fatty acid oxidation, we incubated adipocytes with [^14^C]‐labeled palmitic acid and quantified the release of [^14^C]‐labeled CO_2_. CD146 KO adipocytes displayed a significantly higher rate of fatty acid oxidation (FAO) than WT adipocytes (Figure 5U) alongside the upregulation of FAO‐related genes, such as *Ucp2* and *Acox1* (Figure 5V). Therefore, these results indicate that CD146 KO increased the turnover rate of fatty acids, which likely undergo oxidation after uptake instead of being stored in the form of TGs.

### 2.9 CD146 Interacts with ANGPTL2

**Figure 6:**
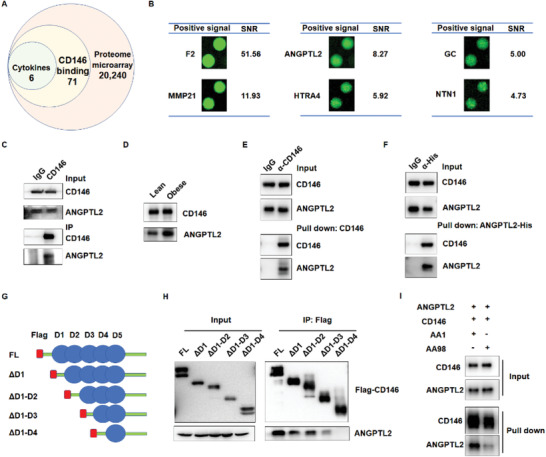
CD146 interacts with ANGPTL2. A) Analysis of CD146‐interacting proteins in the human proteome microarray. B) Fluorescent signal and signal‐noise ratio (SNR) of CD146‐interacting cytokines. C) Co‐immunoprecipitation (co‐IP) assay showing the interaction between CD146 and ANGPTL2 in mouse WAT. D) Co‐IP assay showing the enhanced interaction between CD146 and ANGPTL2 in WAT from obese mice. E,F) Pull‐down assay showing the direct interaction between CD146 and ANGPTL2. Purified His‐tagged ANGPTL2 and CD146 proteins were incubated in PBS. CD146 and ANGPTL2 were pulled down using anti‐CD146 antibodies E) and anti‐His tag antibodies F), respectively. G) Schematic diagram of the full‐length (FL) and CD146 truncates fused with Flag‐tag. H) Co‐IP assay showing that the fourth domain of CD146 is required for the ANGPTL2‐CD146 interaction. ANGPTL2 was coexpressed with different CD146 truncates in 293 cells. I) Pull‐down assay showing that anti‐CD146 AA98 antibodies blocked the CD146‐ANGPTL2 interaction.

Adiponectin is an important adipocytokine that plays multiple roles in lipid metabolism and adipose inflammation.^[^ ^35^ ^]^ Since previous studies have reported that adiponectin expression is correlated with CD146 in several clinical diseases,^[^ ^36^, ^37^, ^38^, ^39^ ^]^ we investigated whether CD146 participates in adiponectin‐mediated lipid metabolism and adipose inflammation. Interestingly, CD146‐mediated obesity downregulated adiponectin expression in a manner that seemed to be related to obesity, but not CD146 (Figure S8A,B, Supporting Information). Moreover, CD146 KO did not affect adiponectin‐mediated lipid uptake, lipogenesis, lipolysis, FAO, or adipose inflammation (Figure S8C–G, Supporting Information). Therefore, we screened for CD146‐interacting proteins using a human proteome microarray (HPM) to identify CD146 ligands that regulate adipogenesis, lipid accumulation, and adipose inflammation. When the HPM was incubated with CD146‐Cy3, 71 of the 20,240 proteins in the microarray showed positive Cy3 fluorescent signals, indicating an interaction with CD146, while six were extracellular factors that included the validated CD146 ligand Netrin‐1 (NTNI; **Figure** **6**A,B; Figure S9A and Table S1, Supporting Information).

Of these top candidate proteins, only ANGPTL2 has been reported to play an important role in obesity and related diseases (Figure 6B) and its adipose receptor has not been identified; therefore, we decided to focus on ANGPTL2. Co‐immunoprecipitation (co‐IP) assays indicated that CD146 interacted with ANGPTL2 in WAT (Figure 6C), but not with other ANGPTL family members such as ANGPTL3 or ANGPTL4 (Figure S9B,C, Supporting Information). In addition, CD146 and ANGPTL2 formed more complexes in WAT from obese mice than from lean mice (Figure 6D). To further explore whether ANGPTL2 interacts directly with CD146, we performed a pull‐down assay using purified ANGPTL2 and CD146 proteins. When the two proteins were coincubated in vitro, ANGPTL2 was pulled down using an anti‐CD146 antibody (Figure 6E). Conversely, CD146 was pulled down using ANGPTL2‐His (Figure 6F). Together, these results suggest that ANGPTL2 and CD146 interact directly with each other.

Next, we mapped the CD146 domain required for the ANGPTL2‐CD146 interaction using Flag‐tagged CD146 truncated variants (Figure 6G). Co‐IP assays indicated that ANGPTL2 interacts with different CD146 variants, namely full length, CD146 with deleted 1st domain (ΔD1), CD146 with deleted 1st and 2nd domains (ΔD1‐2), and CD146 with deleted 1st domain to the 3rd domain (ΔD1‐3). However, ANGPTL2 failed to interact with CD146 containing only the 5th domain (ΔD1‐4; Figure 6H), indicating that the 4th Ig‐like region of CD146 is critical for its binding to ANGPTL2. Previously, we developed two anti‐CD146 antibodies: antibody AA98 recognizes CD146 at its 4th to 5th Ig domain, while antibody AA1 recognizes CD146 at its 1st to 2nd domain.^[^ ^40^, ^41^ ^]^ Using a pull‐down assay, we demonstrated that AA98, but not AA1, blocks the interaction between ANGPTL2 and CD146 (Figure 6I), consistent with our interaction mapping observation. Previously, we reported that CD146 interacts with VEGFR2, CD36, and PDGFR*β* in the endothelium, macrophages, and pericytes, respectively.^[^ ^22^, ^24^, ^42^ ^]^ Here, we observed that these CD146 coreceptors were also expressed in adipocytes (Figure S9D, Supporting Information) and that their knockdown did not affect the interaction between CD146 and ANGPTL2 (Figure S9E–G, Supporting Information).

### 2.10 ANGPTL2‐CD146 Interaction Promotes Adipogenesis, Lipid Accumulation, and Adipose Inflammation

**Figure 7:**
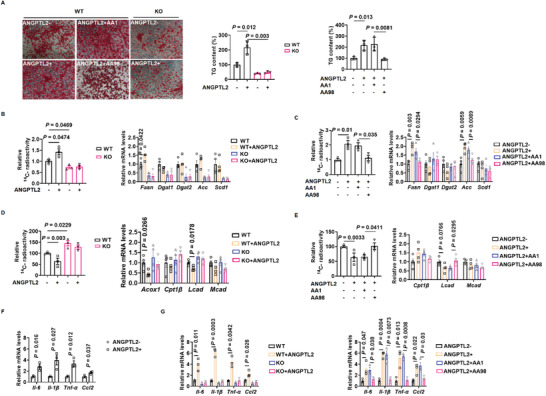
ANGPTL2‐CD146 interaction regulates adipogenesis, lipid metabolism, and adipose inflammation. A) Oil‐red‐O staining and triglyceride (TG) content of adipocytes differentiated from WT and CD146 KO SVF treated with ANGPTL2 and anti‐CD146 AA98 or AA1 antibodies. B) ^14^C radioactivity (left) and lipogenesis‐related gene expression (right) in WT and CD146 KO adipocytes treated with ANGPTL2 (^14^C radioactivity, *n* = 3 per group; gene expression, *n* = 4 per group). C) ^14^C radioactivity (left) and lipogenesis‐related gene expression (right) in adipocytes treated with ANGPTL2 and anti‐CD146 AA98 or AA1 antibodies (^14^C radioactivity, *n* = 3 per group; gene expression, *n* = 4 per group). D) ^14^C radioactivity (left) and fatty acid oxidation‐related gene expression (right) in WT and CD146 KO adipocytes treated with ANGPTL2 (^14^C radioactivity, *n* = 3 per group; gene expression, *n* = 4 per group). E) ^14^C radioactivity (left) and fatty acid oxidation‐related gene expression (right) in adipocytes treated with ANGPTL2 and anti‐CD146 AA98 or AA1 antibodies (^14^C radioactivity, *n* = 3 per group; gene expression, *n* = 4 per group). F) RT‐qPCR analysis of proinflammatory factor *Il‐6*, *Il1‐β*, *Tnf‐α*, and *Ccl2* upregulation in adipocytes treated with ANGPTL2 (*n* = 3 per group). G) RT‐qPCR analysis of proinflammatory factor *Il‐6*, *Il1‐β*, *Tnf‐α*, and *Ccl2* expression in adipocytes treated with ANGPTL2 and anti‐CD146 AA98 or AA1 antibodies (*n* = 3 per group). Data represent the mean ± SD in (A) and mean ± SEM in (B)–(G). *P* values were determined using Student's *t*‐tests in (F) and two‐way ANOVA in (A–E) and (G).

To examine whether the interaction between ANGPTL2 and CD146 is essential for adipogenesis, we isolated SVF from both WT and CD146 KO mice and induced adipocyte differentiation with or without ANGPTL2. Adding ANGPTL2 efficiently promoted the differentiation of WT SVF into adipocytes in a manner dependent on CD146 expression, while ANGPTL2 failed to enhance adipocyte differentiation when CD146 was knocked out (**Figure** **7**A). In addition, the anti‐CD146 antibody AA98, but not AA1, suppressed adipocyte differentiation, consistent with the observation that AA98 blocks the ANGPTL2‐CD146 interaction (Figure 7A). We then isolated mature adipocytes from WT and CD146 KO mice and examined the effect of the ANGPTL2‐CD146 interaction on fatty acid uptake, lipogenesis, lipolysis, and FAO. Although ANGPTL2 did not influence fatty acid uptake (Figure S10A, Supporting Information), it promoted lipogenesis and suppressed FAO in WT adipocytes (Figure 7B–E); however, these effects of ANGPTL2 were abrogated by either CD146 ablation or AA98 treatment, indicating that lipogenesis and FAO regulation by ANGPTL2 requires CD146 (Figure 7B–E). We also observed that ANGPTL2 slightly regulated lipolysis in a CD146‐independent manner (Figure S10B, Supporting Information). Collectively, these data indicate that the ANGPTL2‐CD146 interaction promotes adipogenesis in preadipocytes and lipid accumulation in mature adipocytes by enhancing lipogenesis and suppressing FAO.

Since the ANGPTL2‐CD146 interaction is also critical for adipose inflammation, we isolated visceral adipocytes from WT and CD146 KO mice and stimulated them with ANGPTL2 in vitro. ANGPTL2 stimulation upregulated the expression of proinflammatory factors (IL‐6, IL‐1*β*, TNF‐*α*, and CCL2) in WT visceral adipocytes (Figure 7F), but not in CD146 KO cells (Figure 7G, left). In addition, blocking the ANGPTL2‐CD146 interaction using AA98 antibodies suppressed ANGPTL2‐mediated proinflammatory adipokine upregulation (Figure 7G, right). Together, these results suggest that the ANGPTL2‐CD146 interaction promotes adipogenesis, lipid accumulation, and adipose inflammation, and can be blocked using the anti‐CD146 antibody AA98.

### 2.11 ANGPTL2, CD146, and CREB Form a Positive Feedback Loop

To identify potential downstream factors activated by the interaction between ANGPTL2 and CD146, we tested a series of potential downstream signaling pathways in SVF treated with ANGPTL2. Interestingly, ANGPTL2 activated PKA, CREB, p38, p65, AKT, and ERK in WT SVF, but only PKA and CREB phosphorylation were suppressed when CD146 was deleted (Figure S11A, Supporting Information). Meanwhile, cAMP, the upstream activator of PKA, and C/EBP*β*, the downstream target of CREB, were upregulated in WT SVF but not in KO SVF (Figure S11B,C, Supporting Information). To examine whether ANGPTL2‐mediated CREB activation depended on PKA activation, we treated cells with the PKA inhibitor H‐89, finding that H‐89 suppressed ANGPTL2‐induced PKA and CREB phosphorylation and C/EBP*β* upregulation (Figure S11D,E, Supporting Information). Consistently, AA98 anti‐CD146 antibodies also suppressed ANGPTL2‐induced PKA and CREB phosphorylation and C/EBP*β* upregulation (Figure S11F,G, Supporting Information). Furthermore, we observed that ANGPTL2 increased PKA and CREB phosphorylation and inflammation‐related gene expression in visceral adipose tissue which were inhibited by H‐89 and anti‐CD146 antibody AA98 (Figure S11H–L, Supporting Information). Notably, ANGPTL2 also upregulated CD146 expression, consistent with the observation that CREB directly binds to the CD146 promoter, thus activating CD146 expression (Figure S11M, Supporting Information). Therefore, these findings suggest that ANGPTL2, CD146, and CREB form a positive feedback loop during adipogenesis, lipid accumulation, and adipose inflammation (Figure S11N, Supporting Information).

### 2.12 Galectin‐1 Inhibits UCP1 Expression in BAT via CD146

To investigate whether ANGPTL2 influences BAT function by manipulating UCP1 expression, we treated BAT with ANGPTL2 and examined UCP1 expression, finding that ANGPTL2 did not affect UCP1 expression in BAT (Figure S12A, Supporting Information). Since previous studies have shown that galectin‐1 and galectin‐3 play important roles in obesity and interact with CD146 in the endothelium,^[^ ^43^, ^44^, ^45^, ^46^, ^47^, ^48^, ^49^ ^]^ we examined whether these proteins regulated UCP1 expression in BAT. Interestingly, galectin‐1, but not galectin‐3, inhibited UCP1 expression (Figure S12B,C, Supporting Information) and CD146‐dependent UCP1 expression in BAT (Figure S12D, Supporting Information). Co‐IP assays also revealed that galectin‐1 and CD146 interacted in BAT (Figure S12E, Supporting Information), while pull‐down assays using purified galectin‐1 and CD146 confirmed a direct interaction between these proteins (Figure S12F,G, Supporting Information). In addition, galectin‐1 and CD146 interactions were blocked by AA98 but not AA1 (Figure S12H, Supporting Information), while the galectin‐1‐CD146 interaction was found to downregulate UCP1 in BAT by enhancing AKT and FoxO1 phosphorylation (Figure S12I, Supporting Information), which have been reported to regulate UCP1 expression in BAT.^[^ ^50^, ^51^ ^]^ Taken together, these findings suggest that CD146 suppresses UCP1 expression in BAT via galectin‐1.

### 2.13 Targeting CD146 Prevents Obesity and Insulin Resistance

**Figure 8:**
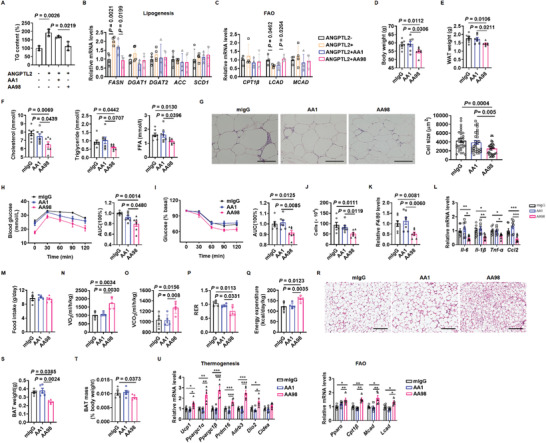
Anti‐CD146 AA98 antibody attenuates obesity development and obesity‐related inflammation. A) Triglyceride (TG) content of adipocytes differentiated from human SVF treated with ANGPTL2 or anti‐CD146 AA98 or AA1 antibodies. RT‐qPCR showing the expression of lipogenesis B) and fatty acid oxidation‐related genes C) in human adipocytes treated with ANGPTL2 or anti‐CD146 AA98 or AA1 antibodies. Body weight D) and WAT weight E) of db/db mice treated with AA98, AA1, or mIgG (*n* = 7 per group). F) Serum cholesterol, triglyceride, and free fatty acid (FFA) levels in db/db mice treated with AA98, AA1, or mIgG (*n* = 7 per group). G) Representative H&E images and quantification of adipocyte size in db/db mice treated with AA98, AA1, or mIgG. H) GTT (left) and the AUC of GTT (right) in db/db mice treated with AA98, AA1, or mIgG (*n* = 7 per group). I) ITT (left) and the AUC of ITT (right) in db/db mice treated with AA98, AA1, or mIgG (*n* = 7 per group). Macrophage number J) and macrophage gene *F4/80* expression K) in the visceral fat pads of db/db mice treated with AA98, AA1, or mIgG (*n* = 7 each group). L) RT‐qPCR showing the expression of proinflammatory factors in visceral adipose tissue from db/db mice treated with AA98, AA1, or mIgG (*n* = 7 per group). M) Food intake of db/db mice treated with AA98, AA1, or mIgG (*n* = 5 per group). Oxygen consumption N), carbon dioxide production O), respiratory quotient P) and energy expenditure Q) of db/db mice treated with AA98, AA1, or mIgG (*n* = 5 per group). R) Representative H&E images of BAT from db/db mice treated with AA98, AA1, or mIgG. Scale bar, 200 µm. BAT weight S) and mass T) of db/db mice treated with AA98, AA1, or mIgG (*n* = 5 each group). U) RT‐qPCR showing the expression of thermogenesis and fatty acid oxidation (FAO)‐related genes in BAT from db/db mice treated with AA98, AA1, or mIgG (*n* = 5 per group). Data represent the mean ± SD in (A)–(C) and mean ± SEM in (D‐U). *P* values were determined using two‐way ANOVA in (A)–(C) and one‐way ANOVA in (D‐U). *P < 0.05, **P < 0.01, ***P < 0.001

Finally, we investigated whether targeting CD146 using the anti‐CD146 AA98 antibody could prevent obesity and its related complications. We examined the effect of AA98 on human adipocyte differentiation in SVF isolated from human WAT by quantifying TG content. Since AA98 efficiently suppressed ANGPTL2‐induced human adipocyte differentiation (**Figure** **8**A), we analyzed the effect of AA98 on lipid accumulation in human mature adipocytes by measuring the expression of lipogenesis and FAO‐related genes. Consistently, ANGPTL2 upregulated lipogenesis‐related genes and downregulated FAO‐related genes in human adipocytes, whereas AA98 strongly reversed these effects (Figure 8B,C).

To determine whether AA98 anti‐CD146 antibodies were able to suppress obesity and related chronic inflammation in vivo, we treated 6 week old male db/db mice with either AA1 or AA98 anti‐CD146 antibodies or control mIgG via intraperitoneal injection twice a week. After 6 weeks, body and WAT weight were significantly reduced in the AA98‐treated group compared to the mIgG control group (Figure 8D,E) whereas AA1 anti‐CD146 antibodies failed to protect the mice from obesity, consistent with the finding that AA98 but not AA1 blocks the interactions between CD146 and its ligands (Figure 8D,E). In addition, AA98‐treated mice displayed smaller adipocytes and lower serum CHO, TG, and FFA levels (Figure 8F,G), as well as enhanced glucose tolerance and insulin sensitivity and lower levels of macrophage infiltration and F4/80 expression in the visceral fat pad compared to mIgG or AA1‐treated mice (Figure 8H–K). Moreover, AA98 treatment downregulated the expression of proinflammatory factors in visceral fat (Figure 8L).

We also investigated the effect of AA98 on energy homeostasis, finding that AA98 did not affect food intake but increased VO_2_, VCO_2_, and energy expenditure, and decreased the respiratory quotient compared to mIgG or AA1 treatment (Figure 8M–Q). In addition, AA98‐treated mice displayed lower BAT weight and mass, alongside fewer multilocular lipid droplets in BAT than mIgG‐ or AA1‐treated mice (Figure 8R–T). Furthermore, AA98 treatment upregulated the expression of genes related to BAT function (Figure 8U), suggesting that AA98 suppresses obesity by enhancing BAT function. Taken together, our findings suggest that AA98 anti‐CD146 antibodies can suppress obesity and related chronic inflammation.

## 3 Discussion

In recent years, increasing attention has been paid to obesity and its related complications.^[^ ^52^ ^]^ Although considerable progress has been made in controlling obesity and many transcription factors and coregulators have been shown to play important roles in this process, few targetable cell membrane receptors have been identified. In this study, we found that CD146 functions as a receptor for ANGPTL2, an important adipose factor secreted by adipocytes. By interacting with ANGPTL2 and another ligand, galectin‐1, CD146 promotes adipogenesis and lipid accumulation and suppresses energy expenditure. Therefore, disrupting the interaction between CD146 and its ligands by CD146 ablation or using anti‐CD146 antibodies efficiently suppresses adipogenesis and increases BAT thermogenesis and lipid turnover in adipocytes by disrupting TG synthesis and enhancing FAO.

Previous studies have reported that ANGPTL2 plays an essential role in insulin resistance and adipose inflammation by acting on endothelial cells and monocytes/macrophages via integrin signaling.^[^ ^13^, ^15^, ^16^, ^53^ ^]^ ANGPTL2 also appears to promote obesity since ANGPTL2 KO mice fail to develop HFD‐induced obesity; however, it remains unclear how ANGPTL2 links adipose inflammation to obesity and whether ANGPTL2 acts directly on adipocytes given that integrin is expressed at low levels in these cells. In this study, we identified CD146 as an ANGPTL2 receptor in adipocytes and found that their interaction promotes obesity and adipose inflammation by enhancing adipogenesis and lipogenesis and suppressing fatty acid oxidation. Therefore, ANGPTL2 can promote obesity and adipose inflammation in multiple ways: 1) ANGPTL2 binds to integrin in the endothelium to promote macrophage infiltration via NF‐*κ*B signaling, 2) ANGPTL2 binds to CD146 to increase inflammatory adipokine (TNF‐*α*, IL‐6, and IL‐1*β*) expression by activating CREB in adipocytes. Notably, CD146 is highly expressed in the endothelial cells of nascent blood vessels;^[^ ^40^ ^]^ therefore, it would be interesting to investigate how ANGPTL2 regulates adipose inflammation or angiogenesis by interacting with CD146 in endothelial cells. ANGPTL2 and CD146 may also have independent functions regulating plasma CHO and TG levels, since it has been reported that ANGPTL2 deficiency does not affect plasma CHO or TG levels in HFD‐fed mice,^[^ ^13^, ^54^ ^]^ whereas CD146 KO reduces their levels. Moreover, ANGPTL2 activates numerous signaling pathways, including PKA, CREB, p38, p65, AKT, and ERK in adipocytes, yet only PKA and CREB activation require CD146. Therefore, ANGPTL2 may exert other roles by interacting with unknown receptors besides CD146.

Although CD146 is required for adipogenesis to activate C/EBP *α* and PPAR*γ* via CREB, CD146 is expressed at low levels in preadipocytes before differentiation begins. This phenomenon can be explained by the positive feedback loop formed by ANGPTL2, CD146, and CREB, wherein ANGPTL2‐CD146 activates CREB, which is essential for upregulating the master regulators C/EBP *α* and PPAR*γ* that initiate adipogenesis. Subsequently, CREB upregulates CD146 expression, which in turn boosts CREB activation. Notably, the ANGPTL2 promoter harbors a CREB binding site^[^ ^55^ ^]^ and is gradually upregulated during adipocyte differentiation.^[^ ^13^, ^53^ ^]^ Thus, is likely that CREB directly upregulates ANGPTL2 expression during adipocyte differentiation, meaning that the feedback loop enhances ANGPTL2 and CD146 expression, which increases obesity and inflammation.

CD146 has previously been reported to function as an oncogene in tumor cells and to promote angiogenesis in endothelial cells.^[^ ^56^ ^]^ In this study, we found that CD146 was highly expressed in mouse WAT and further elevated in WAT from high‐fat diet‐induced or age‐related obesity. In addition, the analysis of clinical samples indicated that CD146 expression correlates positively with the BMI of humans, suggesting that CD146 could be a potential therapeutic target for treating obesity. Indeed, we found that blocking the interaction between CD146 and its ligands using an anti‐CD146 antibody efficiently suppressed the onset of obesity and its related complications. Notably, CD146 expression is low in many other tissues, including the heart, brain, kidney, liver, and muscle; therefore, targeting CD146 could represent a safer approach with fewer side effects than current therapies.

## 4 Experimental Section

##### Antibodies and Reagents

The antibodies and reagents used in this study are listed in Table S2 (Supporting Information).

##### Cell Culture and Treatment

3T3‐L1 preadipocytes were obtained from the National Infrastructure of Cell Line Resource. Human embryonic kidney (HEK293) cells were purchased from the American Type Culture Collection (ATCC). Both cell lines were maintained in Gibco Dulbecco's modified Eagle medium (DMEM) supplemented with 10% fetal bovine serum (FBS). SVF was freshly isolated from mice and maintained in DMEM/F12 containing 10% FBS. All cells were cultured at 37 °C with 5% CO_2_. ANGPTL2 was tagged using 6× His and Strep, expressed in HEK293 cells, and purified using nickel‐nitrilotriacetic acid (Ni‐NTA) and Strep‐Tactin matrix. Anti‐CD146 AA98 and AA1 antibodies were produced from hybridoma developed in the lab. For the receptor blocking test, cells were incubated with AA98 or control AA1 and mIgG (100 ng mL^−1^) 1 h prior to incubation with ANGPTL2 (25 µg mL^−1^).

##### Retroviral Transduction and Stable Cell Line Generation

HEK293 cells were transfected with pQCXIH‐CD146 plasmids encoding CD146 together with helper plasmids. Viral supernatants were collected 48 h after transfection to infect target cells in the presence of 10 µg mL^−1^ polybrene (Yeasen). Stable cell lines were selected in the presence of 100 µg mL^−1^ hygromycin B (InvivoGen).

##### Mice

CD146 constitutive KO mice with a C57BL/6 background were generated as described in Figure S3 (Supporting Information). Adipose‐specific CD146 KO (CD146^AT‐KO^) mice with a C57BL/6 background were generated by crossing CD146^flox/flox^ mice with AdipoQ‐derived Cre mice (Figure S6, Supporting Information). AdipoQ‐derived Cre mice with a C57BL/6 background were a kind gift from Dr. Zengqiang Yuan from the Beijing Institute of Basic Medical Sciences. Ob/ob and db/db mice were obtained from the Department of Laboratory Animal Science, Peking University Health Science Center. All mouse maintenance and procedures were approved by the Biomedical Research Ethics Committee of the Institute of Biophysics, Chinese Academy of Sciences, and were performed according to the relevant ethical regulations regarding animal research. All studies were approved by the Ethics Committee of the Institute of Biophysics, Chinese Academy of Sciences (SYXK2019010).

For diet‐induced obesity, 6 week old male mice were divided randomly and fed either an ND (10% fat) or HFD (60% fat, from Hunan Xeno Life Science Incorporated Company, China) until the end of the experiment. GTT and ITT were measured when the mice were 18 weeks old. Body weight was measured weekly.

For antibody treatment, 8 week old male db/db mice were kept under pathogen‐free conditions and injected intraperitoneally (i.p.) with the anti‐CD146 AA1 or AA98 antibodies or the control mIgG (10 mg kg^−1^ body weight) twice a week for 6 weeks. Body weight was measured weekly.

Body composition was measured using a micro‐CT whole body composition analyzer (Aloka LCT‐200, Hitachi, Japan). Serum CHO, FFA, and TG levels were measured at the Beijing 306 Hospital (Beijing, China). Serum insulin levels were examined by Beijing Kangjiahongyuan Biotechnology Co., Ltd (Beijing, China).

##### Human Tissue Samples

Human adipose tissues were obtained from Beijing 306 Hospital and the 7th Medical Center of PLA General Hospital with informed consent from all donors. All studies were approved by the Ethics Committee of Beijing 306 Hospital, Chinese PLA General Hospital, and the Institute of Biophysics, Chinese Academy of Sciences (2020‐39). The clinical and anthropometric variables are shown in Table S3 (Supporting Information), including sex, age, height, and BMI (kg m^−2^).

##### GTT and ITT

Mice were placed on a fasting regime for 6 h, before receiving an injection of d‐glucose (2 g kg^−1^ body weight, i.p.) or insulin (0.75 U kg^−1^ body weight, i.p.). Blood glucose levels were monitored for 120 min using a glucometer (Accu‐Check, Roche) with samples collected from the tip of the tail vein.

##### RT‐qPCR

Total RNA was isolated using TRIzol reagent (Invitrogen), reverse transcribed into cDNA using random primers, and then subjected to qPCR using ChamQ Universal SYBR qPCR Master regents (Vazyme Biotech Co., Ltd, China) following the manufacturer's instructions. The primers used are listed in Table S4 (Supporting Information).

##### Electron Microscopy

Fresh BAT fragments were fixed in 2.5% glutaraldehyde‐2% paraformaldehyde in PB (pH 7.4) at 4 °C overnight, post‐fixed in 1% osmium tetroxide, dehydrated using a graded ethanol series, and embedded in SPIPON812. Ultrathin sections were mounted on copper grids, stained with uranyl acetate and lead citrate, and then observed under an FEI Tecnai Spirit transmission electron microscope.

##### Adipogenesis

3T3‐L1 cells were cultured in adipogenic medium (DMEM with 10% FBS, 0.25 × 10^−3^ m IBMX, 1 × 10^−3^ m dexamethasone, and 1 µg mL^−1^ insulin) for 2 days and then cultured in adipogenic maintenance medium (DMEM with 10% FBS and 1 µg mL^−1^ insulin) for 4 days, with the medium refreshed every 2 days. Subsequently, the cells were maintained in DMEM with 10% FBS for 2 days. Mouse SVF cell differentiation was performed according to a similar protocol. Briefly, human SVF was cultured in basal preadipocyte culture medium (DMEM with 10% FBS, 66 × 10^−9^ m human insulin, 1 × 10^−9^ m T3, 10 µg mL^−1^ human transferrin, and 50 µg mL^−1^ gentamicin) for 2 days and then cultured in preadipocyte differentiation medium (basal preadipocyte culture media with 0.5 × 10^−3^ m IBMX, 1 × 10^−6^ m dexamethasone, and 1 µg mL^−1^ rosiglitazone) for 12 days, with the medium refreshed every 3 days. Cellular TG content was measured using a TG determination kit.

##### Oil‐Red‐O Staining

Adipocytes differentiated from 3T3‐L1 cells or SVF were washed with PBS and fixed with 10% formaldehyde for 15 min. Oil‐red‐O working solution was prepared by mixing 6 mL of stock buffer (0.5 g oil‐red‐O in 100 mL isopropanol) with 4 mL of distilled water and then filtering through a 0.45 µm filter. After the fixation agent had been removed, cells were cultured with oil‐red‐O working solution for 1 h, carefully washed several times with distilled water, and then observed under a microscope.

##### SVF and Adipocyte Isolation

Six‐to‐seven‐week‐old male mice were sacrificed and their inguinal adipose tissue was collected under sterile conditions, minced, and digested with 1 mg mL^−1^ collagenase for 45 min at 37 °C in DMEM/Ham's F‐12 1:1 (DMEM/F12) containing 1% FBS and antibiotics (100 units mL^−1^ penicillin, 0.1 mg mL^−1^ streptomycin). Digested tissues were centrifuged at 250 × *g* for 5 min and adipocytes were collected from the uppermost layer. The pellet, representing the SVF containing preadipocytes, was resuspended in erythrocyte lysis buffer for 5 min, centrifuged at 500 × *g* for 5 min, and then resuspended in a culture medium consisting of DMEM/F12, 10% FBS, and antibiotics. The cell suspension was filtered through a 25 µm sterile nylon mesh and the cells were cultured in DMEM/F12 containing 10% FBS.

##### Lipid Uptake

^3^H‐oleic acid or ^3^H‐palmitic acid mixed with nonradioactive oleic acid/palmitic acid were dissolved in a defatted BSA solution (173 µmol L^−1^) at a fatty acid to BSA molar ratio of 0.25. The solution was incubated with 30 µL white adipocyte suspension at 37 °C for 5 min and then the lipid uptake assay was stopped by adding 5 mL of ice‐cold stop solution. The final solution was washed through a cell filter in 30 mL ice‐cold incubation buffer. After the cell filter had dried, radioactivity was determined using a scintillation counter.

##### Lipogenesis

The white adipocyte suspension was incubated in a water bath at 37 °C in the presence or absence of ANGPTL2 (5 µg mL^−1^) or adiponectin for 30 min (final volume 0.2 mL) and then incubated with d‐glucose (final concentration 0.14 × 10^−3^ m) containing 37 000 Bq mL^−1^ of ^14^C‐d‐glucose for 90 min. Lipids were extracted using 1 mL hexane and 2‐propanol (3:2 v/v). Solvents were dried under nitrogen gas and radioactivity was quantified using a scintillation counter.

##### Lipolysis

White adipocytes were incubated at 37 °C in 500 µL of KRB (12 × 10^−3^ m HEPES, 121 × 10^−3^ m NaCl, 4.9 × 10^−3^ m KCl, 1.2 × 10^−3^ m MgSO_4_, and 0.33 × 10^−3^ m CaCl_2_) containing 2% fatty acid‐free BSA and 0.1% glucose in the presence or absence of ANGPTL2 (5 µg mL^−1^). Glycerol release was measured using Free Glycerol Reagent (Applygen, China).

##### Fatty Acid Oxidation

White adipocytes were incubated with [U‐^14^C] palmitic acid (7400 Bq mL^−1^) in the presence or absence of ANGPTL2 (5 µg mL^−1^) or adiponectin for 1 h at 37 °C with gentle shaking. The fatty acid oxidation rate was then determined by measuring ^14^CO_2_ production.

##### Co‐Immunoprecipitation

HEK293 cells were cotransfected with plasmids encoding ANGPTL2 and the full‐length or truncated CD146 using Lipofectamine 2000. Epididymal adipose tissues were collected from mice fed an ND or HFD. Transfected HEK293 cells or epididymal adipose tissues were lysed in ice‐cold RIPA lysis buffer (150 × 10^−3^ m NaCl, 50 × 10^−3^ m Tris, pH 7.4, 0.25% sodium deoxycholate, 1% NP‐40, 1 × 10^−3^ m PMSF, protease inhibitor cocktails) for 1 h. After centrifugation at 12 000 × *g* for 10 min, soluble supernatants were precleared using 20 µL protein G PLUS‐agarose (Santa Cruz Biotechnology) for 1 h at 4 °C and then immunoprecipitated with specific antibodies and 25 µL of protein G PLUS‐agarose beads or anti‐FLAG M2 agarose beads (Sigma) overnight at 4 °C. The immunoprecipitants were washed three times with a washing buffer (1 m NaCl, 1% NP‐40, 50 × 10^−3^ m Tris–HCl, pH 7.5) and analyzed by western blotting.

##### Pull‐Down Assay

For the pull‐down assays, purified His‐ANGPTL2 and CD146 protein (200 ng mL^−1^ each) were incubated in PBS for 1 h at 4 °C. After immunoprecipitation, ANGPTL2 and CD146 were measured by western blotting.

For receptor blocking, either anti‐CD146 AA1 or AA98 antibodies were first incubated with CD146 (200 ng mL^−1^) and protein G PLUS‐agarose beads before incubation with ANGPTL2 (200 ng mL^−1^). After immunoprecipitation, CD146 and ANGPTL2 levels were detected by western blotting.

##### Immuno‐Histochemistry

Tissues were fixed overnight with 4% paraformaldehyde (PFA) and H&E staining was performed according to the standard protocol. For immuno‐histochemical analysis, sections were incubated with F4/80 antibodies, followed by a biotin‐conjugated secondary antibody and HRP‐conjugated streptavidin successively. F4/80 expression was detected using DAB solution. Sections were counterstained with hematoxylin and images were obtained using an OLYMPUS BX51 microscope.

##### Luciferase Reporter Assay

The pGL3 firefly luciferase reporters containing a 2 kb CD146 promoter with WT or mutated putative CREB binding sites were generated by PCR and confirmed by sequencing. Firefly luciferase reporters were then transfected together with the pRL‐TK plasmid containing the *Renilla* luciferase reporter gene. Luciferase activity was measured using a Dual Luciferase Assay system (Promega). Firefly luciferase activity was normalized to *Renilla* luciferase.

##### ChIP Assay

ChIP assays were performed using a ChIP Assay Kit, according to the manufacturer's instructions. Briefly, protein‐DNA was crosslinked with 1% formaldehyde for 10 min, sonicated to generate fragments between 200 and 500 bp in length, incubated with 10 µg of anti‐CREB antibody or IgG (as a negative control) overnight, and then immunoprecipitated with protein G PLUS‐agarose. The DNA‐protein crosslink was reversed by heating at 65 °C overnight, followed by digestion with proteinase K at 45 °C for 2 h. DNA was then recovered using a QIAquick PCR purification kit (Qiagen). Real‐time PCR was performed using the following primers: 5′‐CACCTGTCATTGCTCCTTCAACC‐3′ and 5′‐AAGTGAGGAACTCGCCGCTGT‐3′.

##### Protein Array Screening

Protein arrays (HuProtTM Human Proteome Arrays, CDI Laboratories) were first incubated with blocking buffer (3% BSA in PBS) for 3 h at 4 °C and then incubated with 0.03 mg mL^−1^ CD146‐Cy3 in PBST containing 1% BSA overnight at 4 °C. The arrays were then washed with PBST for 4  ×  10 min, washed with deionized water for 2  ×  5 min, and then washed for 2  ×  5 min with deionized water. Finally, the arrays were centrifuged, dried, and scanned.

##### BAT Mitochondrial Isolation

The entire mitochondrial isolation procedure was conducted at 4 °C. Briefly, BAT was dissected and washed in ice‐cold MSHE buffer (70 × 10^−3^ m sucrose, 220 × 10^−3^ m mannitol, 10 × 10^−3^ m KH_2_PO_4_, 5 × 10^−3^ m MgCl_2_, 2.0 × 10^−3^ m HEPES, and 1.0 × 10^−3^ m EGTA, pH 7.2) to remove blood and connective tissue and then minced and homogenized in MSHE supplemented with 0.2% (w/v) fatty acid‐free BSA. The homogenates were then centrifuged at 800 × *g* for 10 min at 4 °C. After fat/lipid had been removed, the remaining supernatant was centrifuged at 8000 × *g* for 10 min, decanted rapidly, and the pellet washed and resuspended in MSHE buffer. Protein concentration was determined using the Bradford assay.

##### BAT Mitochondrial Respiration Assays

Respirometry measurements were performed using a Seahorse XF24 instrument. Briefly, BAT mitochondria were re‐suspended in MAS buffer (70 × 10^−3^ m sucrose, 220 × 10^−3^ m mannitol, 10 × 10^−3^ m KH_2_PO_4_, 5 × 10^−3^ m MgCl_2_, 2 × 10^−3^ m HEPES, 1 × 10^−3^ m EGTA, and 0.2% (w/v) BSA (fatty acid free), adjusted to pH 7.2). Next, 50 µL of the mitochondrial suspension was added to an XF24 cell culture microplate (2.5 µg protein per well), centrifuged at 2000 × *g* for 20 min at 4 °C, and then 450 µL of prewarmed (37 °C) MAS buffer was added to each well. Baseline respiration was measured. After the addition of substrate (pyruvate (10 × 10^−3^ m) and malate (5 × 10^−3^ m)), substrate‐induced respiration was measured. UCP1‐dependent respiration was assessed by the addition of 2 × 10^−3^ m guanosine diphosphate (GDP).

##### Statistical Analysis

Statistical significance was calculated using GraphPad prism 8 software (GraphPad Inc., San Diego, CA, USA). Animal and clinical data are shown as the mean ± SEM, other data are shown as the mean ± SD. Statistical analyses between two groups were carried out using Student's *t*‐tests. Comparisons between groups involving three mouse genotypes were assessed using one‐way ANOVA. Comparisons between groups involving two mouse genotypes treated with different antibodies were assessed using two‐way ANOVA. *P*‐values of <0.05 were considered statistically significant.

## Author Contributions

Z.W. and J.L. contributed equally to this work. Z.W., X.Y., J.F., and P.B. designed the experiments and analyzed the data. Z.W. and P.B. wrote the manuscript. Z.W. and J.L. performed the majority of the experiments. G.Y., Y.L., and Y.L. assisted with in vitro biochemical analyses. X.C. contributed toward the purification of proteins and antibodies. L.Z. and H.D. contributed toward the fractionation of adipose tissue. Z.L., G.S., and S.Y. assisted with the animal experiments. H.Z. assisted with the radioisotope experiments. L.S. contributed toward the preparation of the TEM sample. G.C., J.D., H.C., H.S., and Y.C. contributed toward the collection of clinical samples. T.W. and P.L. contributed toward BAT mitochondrial respiration data analysis.

## Conflict of Interest

The authors declare no conflict of interest.

## Supporting information

Supporting Information
