# Supplementary material for: CD146 is a Novel ANGPTL2 Receptor that Promotes Obesity by Manipulating Lipid Metabolism and Energy Expenditure
Source: Adv Sci (Weinh). 2021 Jan 27;8(6):2004032. doi: 10.1002/advs.202004032 (PMC7967059; doi:10.1002/advs.202004032)
Supplement: Supplementary file 1 — Supporting Information [file ADVS-8-2004032-s001.pdf]

© 2020 Wiley-VCH GmbH

ADVANCED SCIENCE
Open Access

# Supporting Information

for *Adv. Sci.,* DOI: 10.1002/advs.202004032

# CD146 is a novel ANGPTL2 receptor that promotes obesity by manipulating lipid metabolism and energy expenditure

*Zhenzhen Wu, Jingyu Liu, Gang Chen, Junfeng Du, Huiyun Cai, Xuehui Chen, Gaoqi Ye, Yongting Luo, Yiyi Luo, Liwen Zhang, Hongxia Duan, Zheng Liu, Sai Yang, Hongwei Sun, Yan Cui, Lei Sun, Hongjie Zhang, Guizhi Shi, Taotao Wei, Pingsheng Liu, Xiyun Yan*, Jing Feng*, and Pengcheng Bu**

**Supporting Information**

# CD146 is a novel ANGPTL2 receptor that promotes obesity by manipulating lipid metabolism and energy expenditure

*Zhenzhen Wu, Jingyu Liu, Gang Chen, Junfeng Du, Huiyun Cai, Xuehui Chen, Gaoqi Ye, Yongting Luo, Yiyi Luo, Liwen Zhang, Hongxia Duan, Zheng Liu, Sai Yang, Hongwei Sun, Yan Cui, Lei Sun, Hongjie Zhang, Guizhi Shi, Taotao Wei, Pingsheng Liu, Xiyun Yan*, Jing Feng*, and Pengcheng Bu**

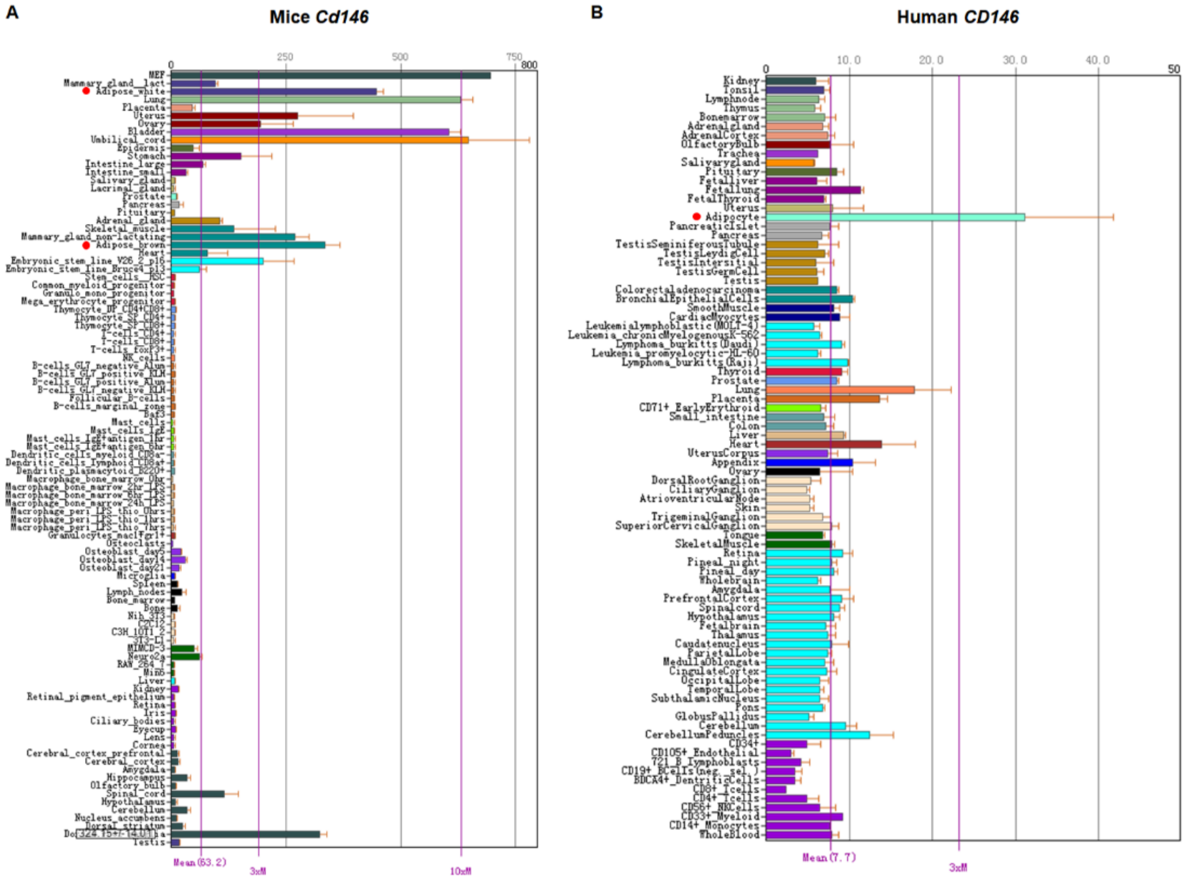


**Figure S1. CD146 is highly expressed in adipose tissues. (A)** BioGPS database showing CD146 expression in mouse adipose tissues. (**B**) BioGPS data base showing CD146 expression in human adipose tissues. Red dots indicate adipose.

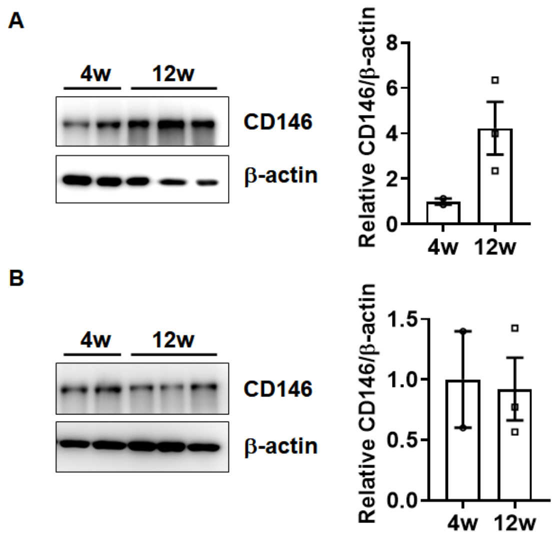


**Figure S2. CD146 expression is upregulated in adipocytes from obese mice.** (**A**) CD146 expression in adipocytes isolated from the WAT of 4-week-old (4w) and 12-week-old (12w) ob/ob mice. (**B**) CD146 expression in SVF isolated from the WAT of 4w and 12w ob/ob mice. All data represent the mean ± SEM. *P* values were determined using Student's *t*-tests.

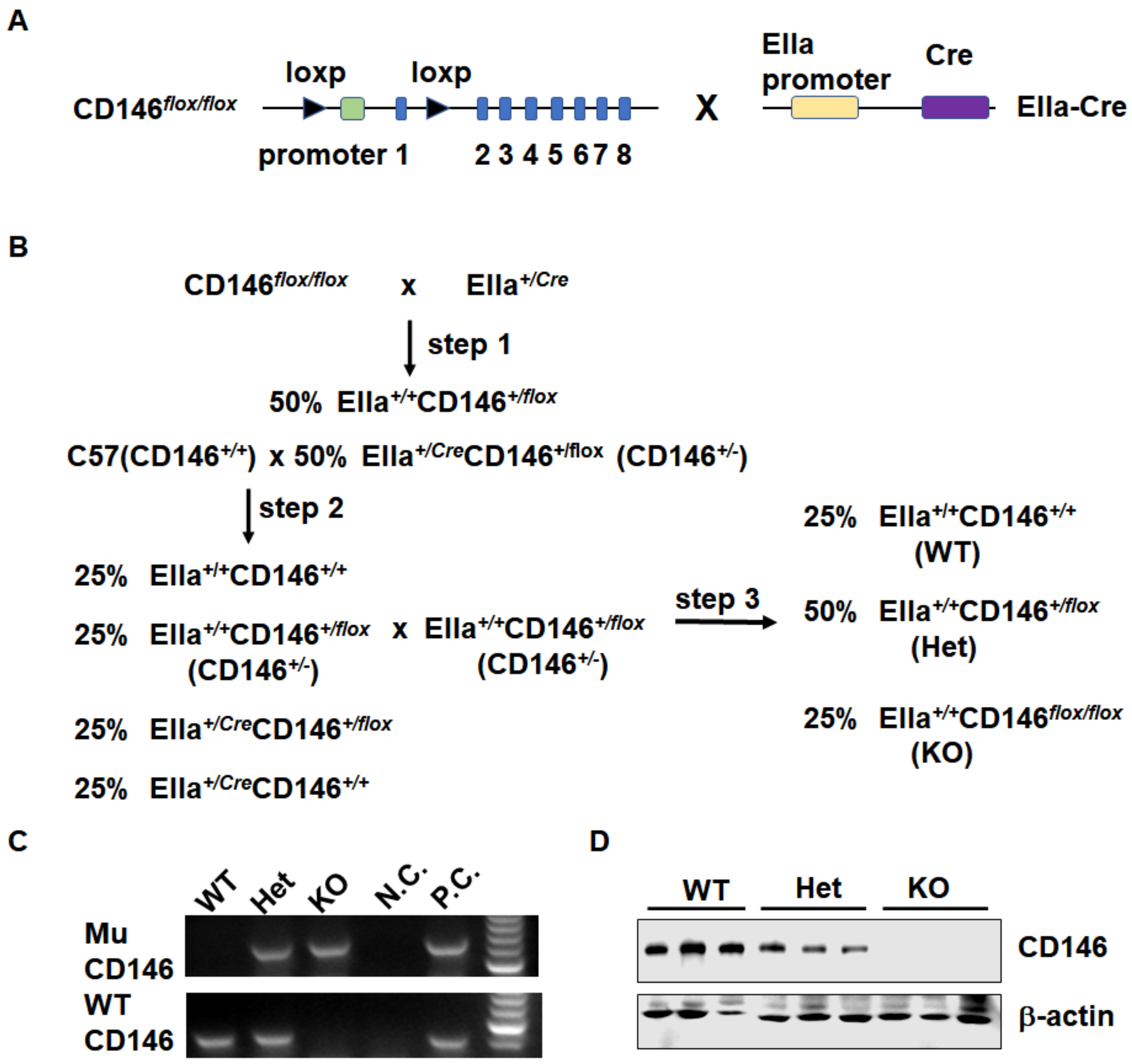


**Figure S3. Generation of CD146 knockout mice.** (**A**) Schematic diagram showing the generation of CD146 knockout (KO) mice by crossing EIIa-*Cre* and CD146$^{flox/flox}$ mice. Loxp sites were inserted upstream of the CD146 promoter and downstream of exon 1, respectively. (**B**) Mating scheme showing the steps used to generate CD146 knockout (KO), heterozygous knockout (Het) and control WT littermate (WT) mice. (**C**) Genotyping of KO, Het, and WT mice. Fragments of the wild-type CD146 gene (WT CD146) and floxed CD146 gene (Mu CD146) were amplified using PCR with specific primers. P.C., positive control; N.C., negative control. (**D**) Western blot showing CD146 expression in WAT from WT, Het, and KO mice.

**A**

| | WT ( n = 5) | Het (n = 5) | KO (n = 5) |
|---|---|---|---|
| Heart (mg) | 170.02 ± 10.83 | 169.10 ± 12.99 | 159.36 ± 8.27 |
| Liver (mg) | 1363.50 ± 56.29 | 1329.76 ± 120.91 | 1216.32 ± 106.87 |
| Spleen (mg) | 101.36 ± 3.15 | 100.44 ± 3.99 | 100.24 ± 2.63 |
| Lung (mg) | 167.93 ± 8.24 | 165.48 ± 5.29 | 154.90 ± 2.89 |
| Left kidney (mg) | 203.66 ± 8.83 | 203.38 ± 9.29 | 200.93 ± 11.05 |

**B**

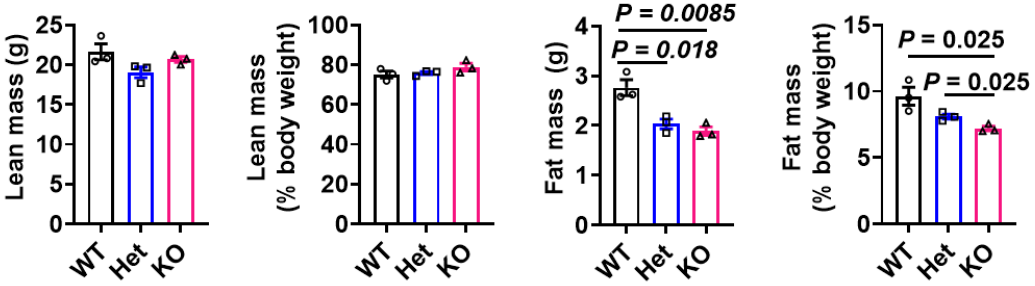


**Figure S4. CD146 KO mice display reduced body weight when fed a normal diet.** (**A**) Weight of various tissues collected from WT, CD146 Het, and KO mice fed a normal diet (ND) (*n* = 5 per group). (**B**) Lean and fat mass of WT, CD146 Het, and KO mice fed a ND (*n* = 3 per group). Data represent the mean ± SEM. *P* values were determined using one-way ANOVA.

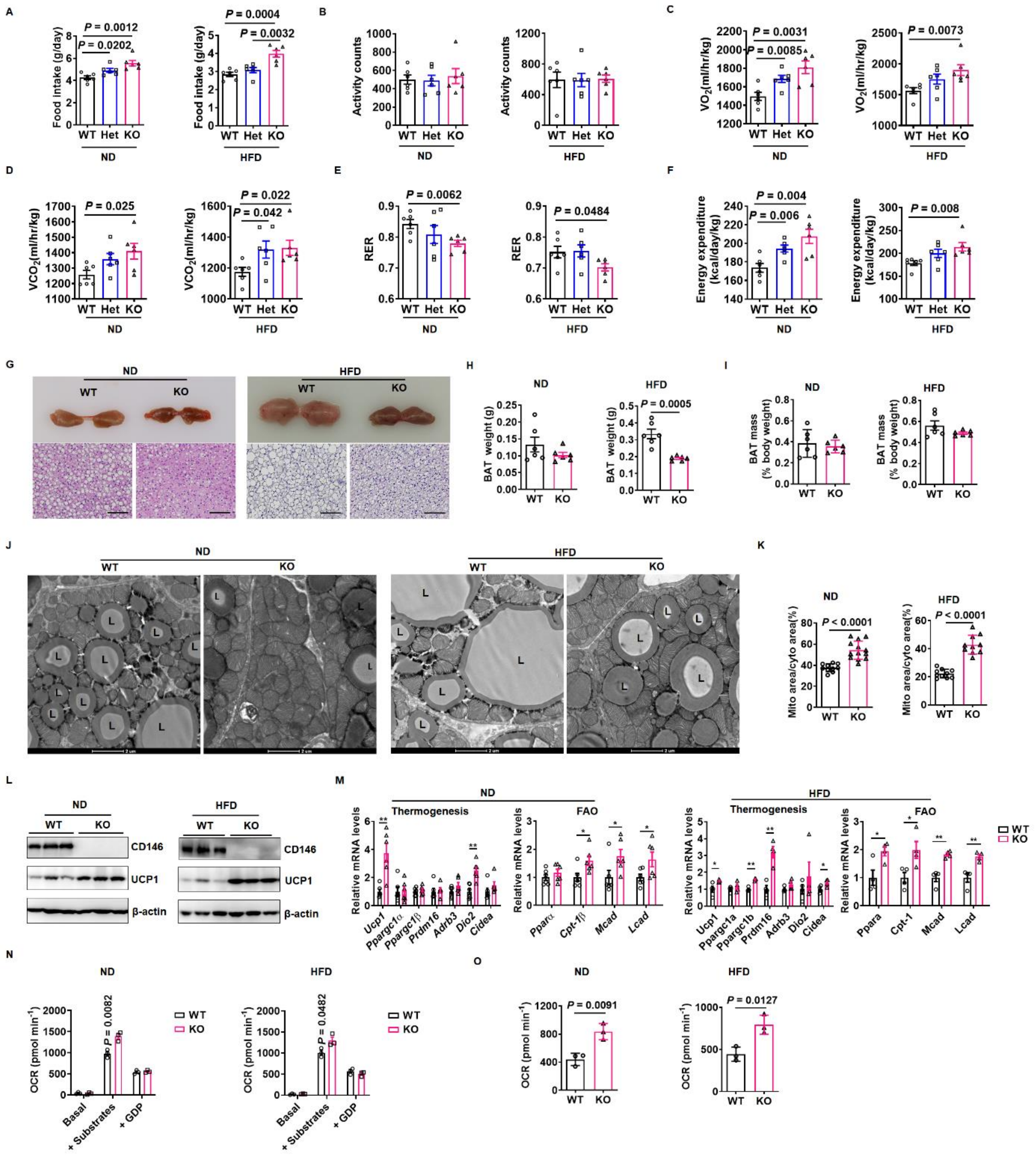


**Figure S5. CD146 knockout enhances energy expenditure and BAT function.** (**A**) Food intake of WT, Het, and KO mice measured daily after 12 weeks of a ND or HFD (*n* = 6 per group). (**B**) Locomotor activity of WT, Het, and KO mice fed a ND or HFD (*n* = 6 per group). (**C-F**) Oxygen consumption (C), carbon dioxide production (D), respiratory quotient (E), and energy expenditure (F) of WT, Het, and KO mice fed a ND or HFD (*n* = 6 per group). (**G**) Representative images (top) and H&E staining (bottom) of BAT from WT and KO mice fed a

ND or HFD. (**H** and **I**) BAT weight (H) and mass (I) of WT and KO mice fed a ND or HFD ($n$ = 6 per group). (**J**) Representative images of BAT mitochondria from WT and KO mice fed a ND or HFD. Scale bar, 2 μm. (**K**) Ratio of mitochondria to cytosol area in WT and KO mice fed a ND or HFD. (**L**) Western blot showing UCP1 expression in BAT from WT and KO mice fed a ND or HFD. (**M**) RT-qPCR analysis of the expression of thermogenesis and fatty acid oxidation (FAO)-related genes in BAT from WT and KO mice fed a ND or HFD (ND, $n$ = 6 per group; HFD, $n$ = 4 per group). (**N**) Oxygen consumption rate (OCR) in mitochondria purified from WT and KO BAT. (**O**) UCP1-dependent respiration calculated based on (N). Data represent the mean ± SEM. $P$ values were determined using one-way ANOVA in (A-F) and Student's $t$-tests in (H, I, K and M-O). $^*P < 0.05$, $^{**}P < 0.01$.

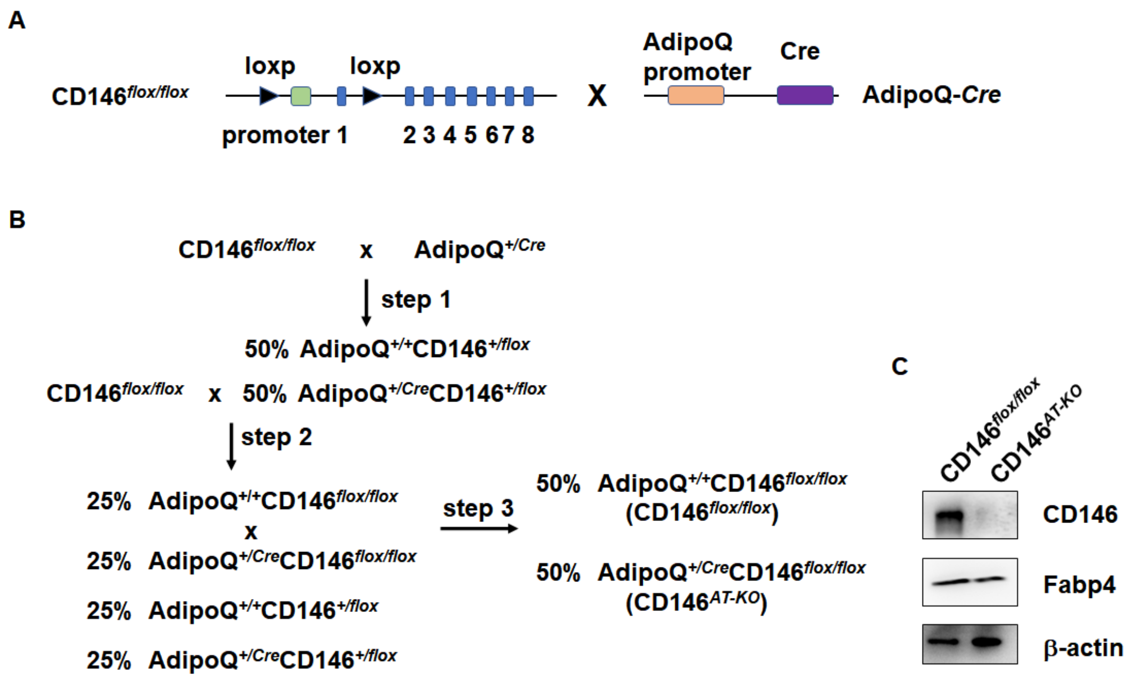


**Figure S6. Generation of adipocyte-specific CD146 knockout mice.** (**A**) Schematic diagram showing the generation of adipocyte-specific CD146 knockout mice (CD146$^{AT\text{-}KO}$) by crossing AdipoQ-*Cre* and CD146$^{flox/flox}$ mice. (**B**) Mating scheme to generate CD146$^{AT\text{-}KO}$ and CD146$^{flox/flox}$ control littermates. (**C**) Western blot showing CD146 expression in CD146$^{flox/flox}$ and CD146$^{AT\text{-}KO}$ mice.

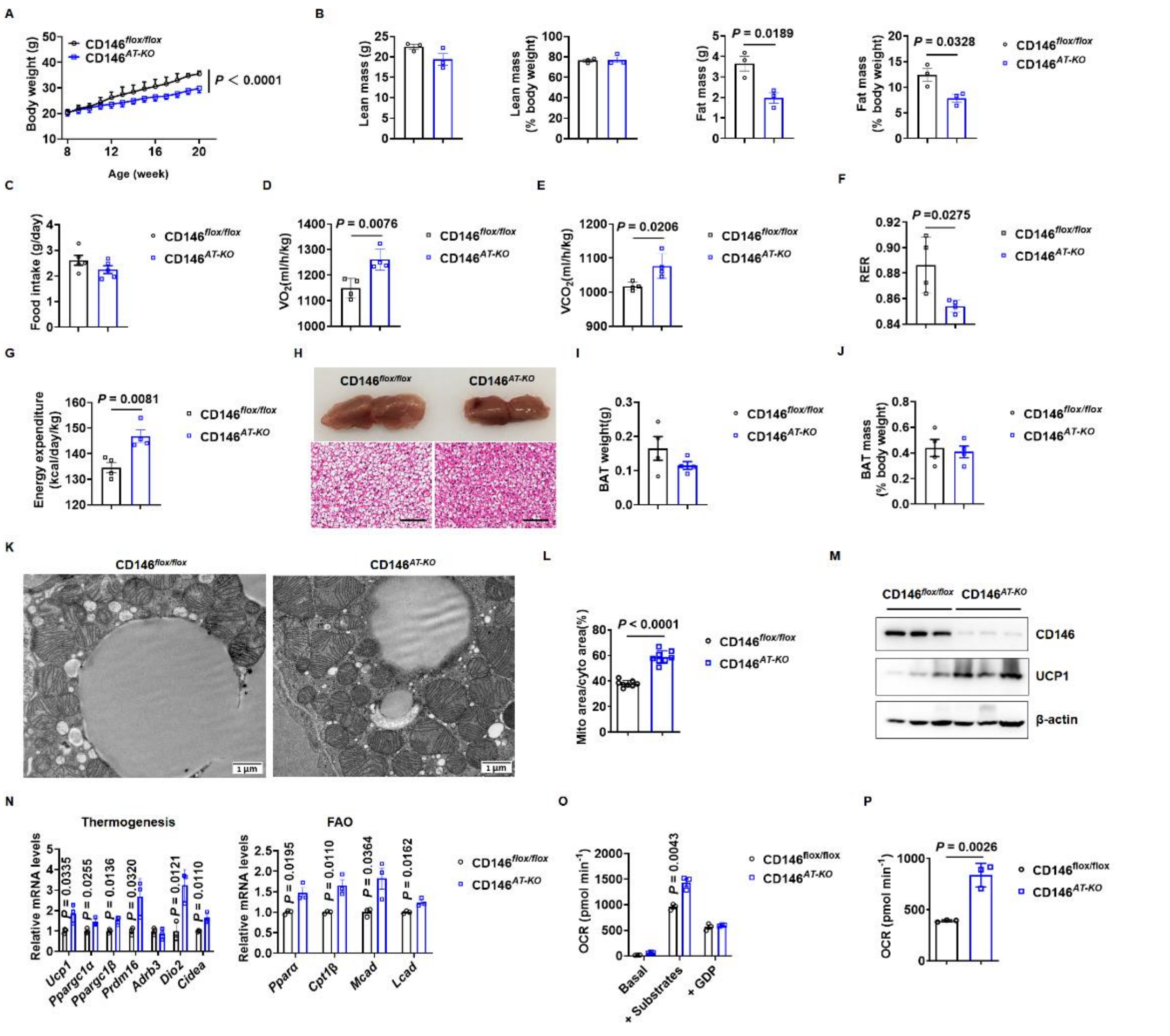


**Figure S7. Adipocyte-specific CD146 knockout enhances energy expenditure and BAT function.** (**A**) Body weight of CD146$^{AT\text{-}KO}$ and CD146$^{flox/flox}$ control mice fed a ND (*n* = 6 per group). (**B**) Lean and fat mass of CD146$^{AT\text{-}KO}$ and CD146$^{flox/flox}$ control mice fed a ND (*n* = 3 per group). (**C**) Food intake of CD146$^{AT\text{-}KO}$ and CD146$^{flox/flox}$ control mice fed a ND (*n* = 5 per group). (**D-G**) Oxygen consumption (D), carbon dioxide production (E), respiratory quotient (F), and energy expenditure (G) of CD146$^{AT\text{-}KO}$ CD146$^{flox/flox}$ control mice fed a ND (*n* = 4 per group). (**H**) Representative images (top) and H&E staining (bottom) of BAT from CD146$^{AT\text{-}KO}$ and CD146$^{flox/flox}$ control mice fed a ND. (**I** and **J**) BAT weight (I) and mass (J) of CD146$^{AT\text{-}KO}$ and CD146$^{flox/flox}$ control mice fed a ND (*n* = 4 per group). (**K**) Representative images of BAT mitochondria from CD146$^{AT\text{-}KO}$ and CD146$^{flox/flox}$ control mice. Scale bar, 1 μm. (**L**) Ratio of

mitochondria to cytosol area in CD146$^{AT\text{-}KO}$ and CD146$^{flox/flox}$ control mice fed a ND. (**M**) Western blot showing UCP1 expression in BAT from CD146$^{AT\text{-}KO}$ and CD146$^{flox/flox}$ control mice fed a ND. (**N**) RT-qPCR analysis of the expression of thermogenesis and fatty acid oxidation (FAO)-related genes in BAT from CD146$^{AT\text{-}KO}$ and CD146$^{flox/flox}$ control mice fed a ND ($n$ = 3 per group). (**O**) Oxygen consumption rate (OCR) in BAT mitochondrion purified from CD146$^{AT\text{-}KO}$ and CD146$^{flox/flox}$ control mice ($n$ = 3 per group). (**P**) UCP1-dependent respiration was calculated based on (O). Data represent the mean ± SEM. *P* values were determined using Student's *t*-tests.

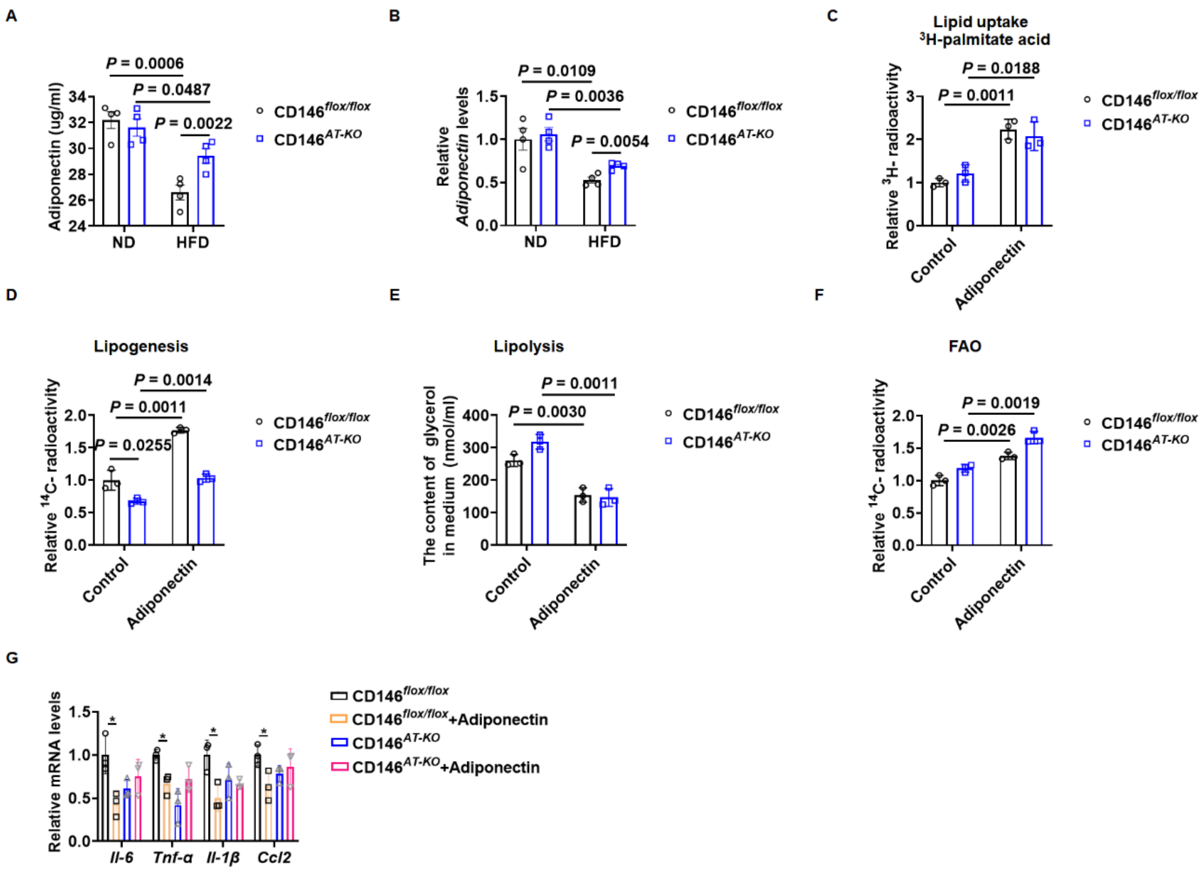


**Figure S8. Correlation between CD146 and adiponectin expression in mice.** (**A**) Serum adiponectin levels in CD146*flox/flox* and CD146*AT-KO* mice fed a ND or HFD (*n* = 4 per group). (**B**) RT-qPCR analysis of adiponectin expression in the adipose tissue of CD146*flox/flox* and CD146*AT-KO* mice fed a ND or HFD (*n* = 4 per group). (**C**) $^{3}H$ radioactivity indicating lipid uptake in CD146*flox/flox* and CD146*AT-KO* adipocytes treated with adiponectin (*n* = 3 per group). (**D**) $^{14}C$ radioactivity indicating lipogenesis in CD146*flox/flox* and CD146*AT-KO* adipocytes treated with adiponectin (*n* = 3 per group). (**E**) Glycerol release indicating the lipolysis rate in CD146*flox/flox* and CD146*AT-KO* adipocytes treated with adiponectin (*n* = 3 per group). (**F**) $^{14}C$ radioactivity indicating fatty acid oxidation (FAO) in CD146*flox/flox* and CD146*AT-KO* treated with adiponectin (*n* = 3 per group). (**G)** RT-qPCR analysis of the expression of inflammation-related genes in CD146*flox/flox* and CD146*AT-KO* adipocytes treated with adiponectin (*n* = 3 per group). Data represent the mean ± SEM. *P* values were determined using two-way ANOVA.

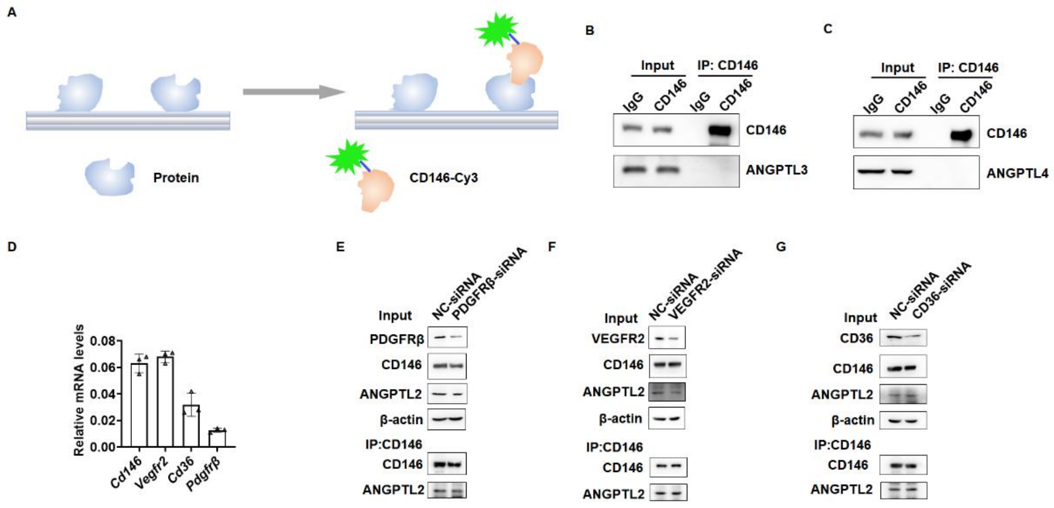


**Figure S9. Identification of proteins directly interacting with CD146.** (**A**) Schematic diagram of the proteome microarray strategy used to identify direct protein-protein interactions. (**B** and **C**) Co-immunoprecipitation assay showing no interaction between CD146 and ANGPTL3 (B) or ANGPTL4 (C). (**D**) RT-qPCR analysis of *Cd146*, *Vegfr2*, *Cd36*, and *Pdgfrβ* expression in adipocytes. (**E-G**) Co-immunoprecipitation assay showing the effect of PDGFRβ (E), VEGFR2 (F), or CD36 (G) knockdown on the interaction between CD146 and ANGPTL2. Data represent the mean ± SD.

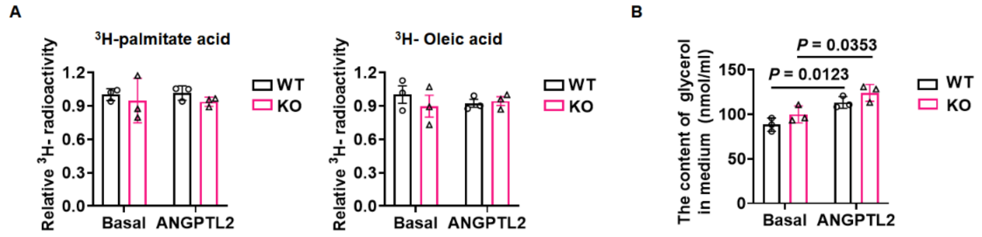


**Figure S10. Regulation of lipid uptake and lipolysis by ANGPTL2.** (**A**) Lipid uptake in WT and CD146 KO adipocytes treated with ANGPTL2 ($n$ = 3 per group). (**B**) Glycerol release indicating the lipolysis rate in WT and CD146 KO adipocytes treated with ANGPTL2 ($n$ = 3 per group). Data represent the mean ± SEM. $P$ values were determined using two-way ANOVA.

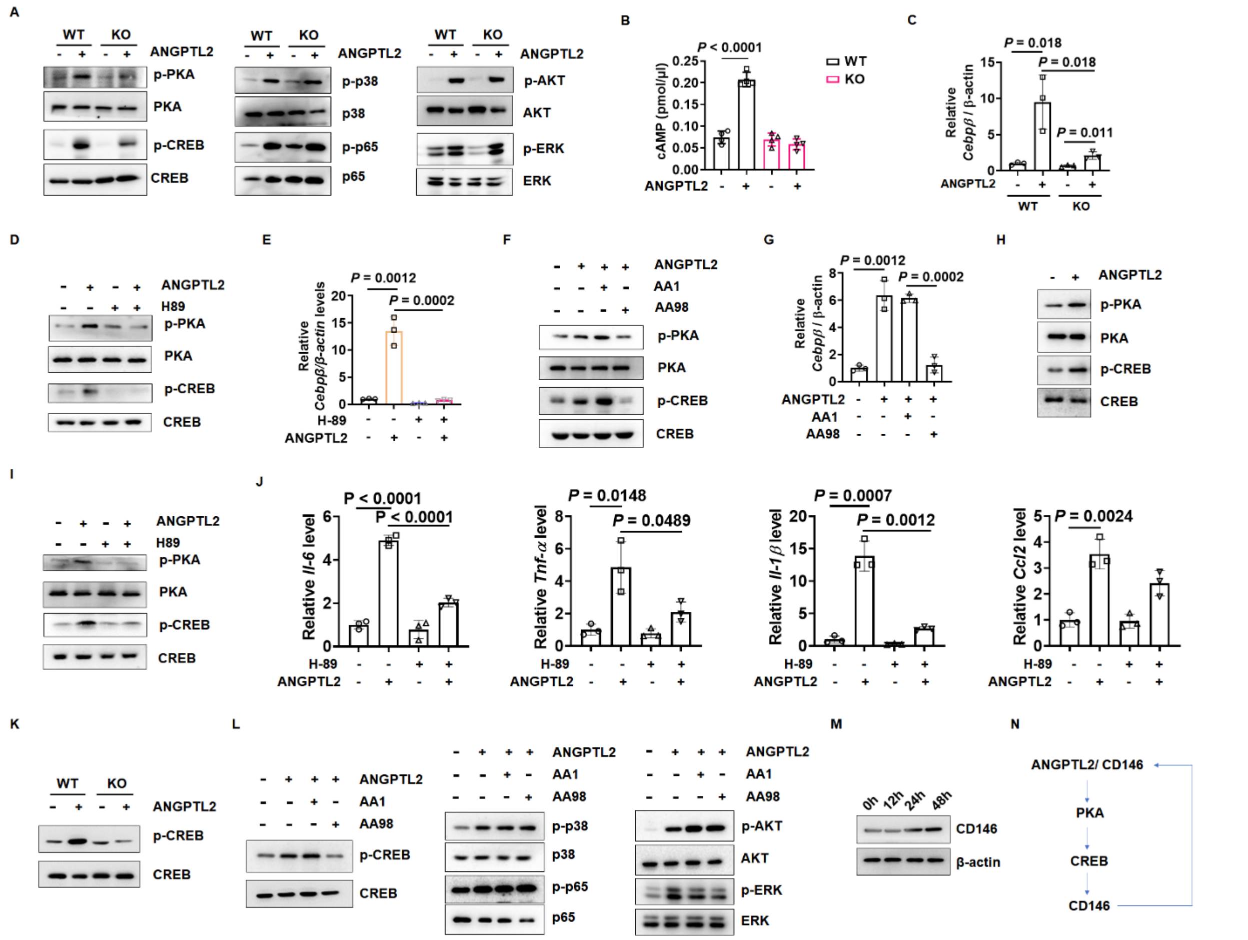

**Figure S11. ANGPTL2, CD146, and CREB form a positive feedback loop.** (**A**) Western blot showing PKA, CREB p38, p65, AKT, and ERK phosphorylation in WT and CD146 KO SVF treated with ANGPTL2. (**B**) Intracellular cAMP levels in WT and CD146 KO SVF treated with ANGPTL2. (**C**) RT-qPCR analysis of *Cebp/β* expression in WT and CD146 KO SVF treated with ANGPTL2. (**D** and **E**) CREB phosphorylation (D) and *Cebpβ* expression (E) in WT and CD146 KO SVF treated with ANGPTL2 and the PKA inhibitor H-89. (**F**) Western blot showing PKA and CREB phosphorylation in SVF treated with ANGPTL2 and anti-CD146 AA98 or AA1 antibodies. (**G**) RT-qPCR analysis of *Cebp/β* expression in SVF treated with ANGPTL2 and anti-CD146 AA98 or AA1 antibodies. (**H**) Western blot showing PKA and CREB phosphorylation in visceral adipose cells treated with ANGPTL2. (**I-J**) CREB phosphorylation (I) and inflammation-related gene expression (J) in visceral adipose cells treated with ANGPTL2 and the PKA inhibitor H-89. (**K**) Western blot showing CREB phosphorylation in WT and CD146 KO visceral adipose cells treated with ANGPTL2. (**L**) Western blot showing CREB

phosphorylation in visceral adipose cells treated with ANGPTL2 and anti-CD146 AA98 or AA1 antibodies. (**M**) Western blot showing that ANGPTL2 upregulates CD146 expression. (**N**) Schematic diagram of the positive feedback loop between ANGPTL2, CD146, and CREB. Data represent the mean ± SD of three independent experiments. *P* values were determined using two-way ANOVA.

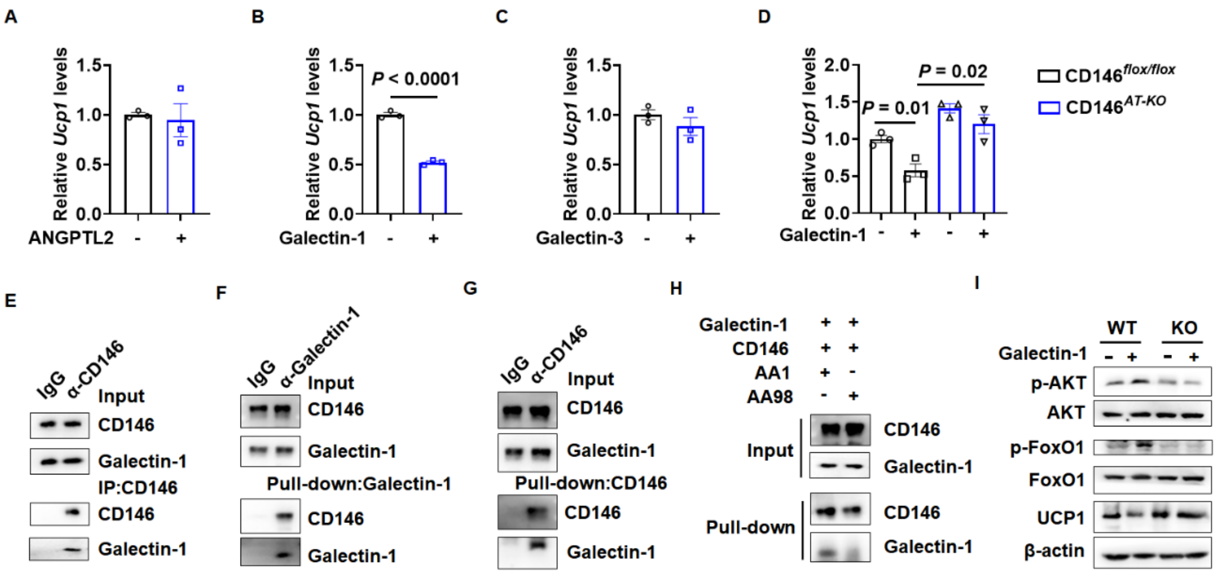


**Figure S12. The galectin-1-CD146 interaction downregulates UCP1 expression in BAT.** (**A**) RT-qPCR analysis of *Ucp*1 expression in BAT with or without ANGPTL2 stimulation (*n* = 3 per group). (**B**) RT-qPCR analysis of Ucp1 expression in BAT with or without galectin-1 stimulation (*n* = 3 per group). (**C**) RT-qPCR analysis of Ucp1 expression in BAT with or without galectin-3 stimulation (*n* = 3 per group). (**D**) RT-qPCR analysis of Ucp1 expression in CD146$^{flox/flox}$ control BAT and CD146$^{AT\text{-}KO}$ BAT with or without galectin-1 stimulation (*n* = 3 per group). (**E**) Co-immunoprecipitation assay showing the interaction between CD146 and galectin-1 in mouse BAT. (**F** and **G**) Pull-down assay showing the direct interaction between CD146 and galectin-1. Galectin-1 and CD146 were pulled down using anti-galectin-1 (F) and anti-CD146 (G) antibodies, respectively. (**H**) Pull-down assay showing that anti-CD146 AA98 antibodies block the CD146-galectin-1 interaction. (**I**) Western blot showing AKT and FoxO1 phosphorylation and UCP1 expression in CD146$^{flox/flox}$ control BAT and CD146$^{AT\text{-}KO}$ BAT stimulated with galectin-1. Data represent the mean ± SEM. *P* values were determined using Student's *t*-tests in (A-C) and two-way ANOVA in (D).

**Table S1. CD146-interacting proteins identified using the proteome array**

| Block | Column | Row | Name | SNR |
|---|---|---|---|---|
| 6 | 16 | 49 | KCNAB2 | 117.00 |
| 13 | 28 | 33 | KCNAB1 | 84.21 |
| 13 | 10 | 9 | CPOX | 74.44 |
| 13 | 18 | 33 | F2 | 51.56 |
| 5 | 8 | 13 | EWSR1 | 32.01 |
| 5 | 16 | 49 | PRLHR | 19.89 |
| 3 | 15 | 45 | RPLP0 | 19.27 |
| 2 | 12 | 45 | FAM103A1 | 16.76 |
| 15 | 29 | 21 | LRRC1 | 15.14 |
| 11 | 9 | 11 | ZADH2 | 13.52 |
| 13 | 23 | 42 | NUPL2 | 12.98 |
| 19 | 1 | 45 | FAM98A | 12.33 |
| 13 | 31 | 7 | FOXM1 | 12.32 |
| 19 | 11 | 54 | MMP21 | 11.93 |
| 6 | 1 | 50 | RBMY1A1 | 12.00 |
| 13 | 9 | 23 | HNRNPD | 11.68 |
| 18 | 16 | 35 | DAZ2 | 11.11 |
| 9 | 1 | 9 | FBXO2 | 10.35 |
| 10 | 21 | 37 | KRTAP20-1 | 8.82 |

| | | | | |
|---|---|---|---|---|
| 8 | 20 | 51 | ANGPTL2 | 8.27 |
| 5 | 8 | 7 | PARL | 7.82 |
| 11 | 2 | 55 | KRTAP19-7 | 7.60 |
| 5 | 2 | 33 | DCDC2 | 7.30 |
| 21 | 10 | 23 | KRTAP6-2 | 7.27 |
| 4 | 19 | 17 | RTP4 | 7.26 |
| 5 | 6 | 50 | WDR53 | 7.15 |
| 16 | 28 | 36 | ARHGEF3 | 7.01 |
| 1 | 11 | 57 | SMRP1 | 6.55 |
| 20 | 29 | 19 | TIAL1 | 6.54 |
| 8 | 16 | 56 | KCNS3 | 6.51 |
| 6 | 3 | 27 | SNX15 | 6.37 |
| 8 | 16 | 52 | TMPO | 6.08 |
| 5 | 32 | 46 | DNAJB6 | 5.74 |
| 21 | 24 | 48 | CIRBP | 5.71 |
| 7 | 30 | 42 | APOBEC3B | 5.63 |
| 2 | 28 | 7 | ABI1 | 5.51 |
| 15 | 2 | 15 | HTRA4 | 5.92 |
| 8 | 1 | 49 | GOSR2 | 5.37 |
| 15 | 1 | 55 | CPT1A | 5.30 |
| 5 | 6 | 30 | ELAVL4 | 5.17 |

| | | | | |
|---|---|---|---|---|
| 1 | 19 | 55 | PSMA3 | 5.08 |
| 10 | 20 | 55 | KRTAP13-1 | 5.06 |
| 21 | 16 | 52 | GC | 5.00 |
| 18 | 13 | 2 | NME4 | 4.95 |
| 16 | 17 | 25 | FAM220A | 4.83 |
| 16 | 25 | 9 | NTN1 | 4.73 |
| 7 | 31 | 57 | FAM49B | 4.66 |
| 17 | 7 | 46 | DUSP26 | 4.65 |
| 6 | 20 | 27 | HSD17B7 | 4.41 |
| 4 | 23 | 43 | BCS1L | 4.31 |
| 23 | 7 | 15 | CIRBP | 4.24 |
| 9 | 1 | 5 | TMEM143 | 4.13 |
| 2 | 24 | 21 | RBFOX2 | 4.03 |
| 15 | 30 | 35 | FAM57A | 3.91 |
| 1 | 19 | 43 | DCTN2 | 3.88 |
| 7 | 12 | 17 | FAM3A | 3.86 |
| 18 | 23 | 9 | CCR7 | 3.54 |
| 18 | 19 | 12 | SLC25A30 | 3.54 |
| 6 | 4 | 31 | CNIH2 | 3.53 |
| 8 | 16 | 54 | SENP3 | 3.50 |
| 15 | 27 | 27 | COX10 | 3.44 |

| | | | | |
|---|---|---|---|---|
| 6 | 30 | 57 | VGLL4 | 3.43 |
| 5 | 20 | 9 | MTG2 | 3.35 |
| 18 | 13 | 23 | LETM1 | 3.34 |
| 11 | 11 | 22 | DNAJB8 | 3.25 |
| 20 | 9 | 29 | SCAMP4 | 3.21 |
| 8 | 6 | 41 | TVP23C | 3.18 |
| 13 | 5 | 37 | GBAS | 3.10 |
| 12 | 22 | 47 | RTP3 | 3.07 |
| 5 | 7 | 30 | FAM131C | 3.05 |
| 5 | 16 | 54 | IL18RAP | 3.01 |

**Table S2. Reagent information**

| Reagent | Source | Identifier |
|---|---|---|
| **Antibodies** | | |
| Anti-mouse CD146 | BD Biosciences | Clone ME-9F1 |
| Rabbit IgG | Cell Signaling | Cat# 9244 |
| Mouse IgG | Sigma | Cat# I8765 |
| Anti-phospho-AKT | Cell Signaling | Cat #4060 |
| Anti-AKT | Cell Signaling | Cat #2920 |
| Anti-phospho-ERK | Cell Signaling | Cat #4370 |
| Anti-ERK | Cell Signaling | Cat #4695 |
| Anti-phospho-CREB | Cell Signaling | Cat #9198 |
| Anti-CREB | Cell Signaling | Cat #9104 |
| Anti-phospho-PKA | Santa Cruz | Cat #sc-377575 |
| Anti-PKA | Santa Cruz | Cat #sc-28315 |
| Anti-phospho-p38 | Cell Signaling | Cat #9218 |
| Anti-p38 | Cell Signaling | Cat #9228 |
| Anti-phospho-p65 | Cell Signaling | Cat #3033 |
| Anti-p65 | Cell Signaling | Cat #6956 |
| Anti-Fabp4 | Cell Signaling | Cat #3544 |
| Anti-C/EBPa | Cell Signaling | Cat #8178 |
| Anti-ANGPTL2 | Abcam | Cat # ab35574 |
| Anti-F4/80 | Abcam | Cat#ab6640 |
| Anti-β-actin | ABGENT | Cat#AM1021b |
| Anti-FoxO1 | Santa Cruz | Cat#sc-11350 |
| Anti-phospho-FoxO1 | Immunoway | Cat#YP0113 |
| Anti-PDGFRβ | Abcam | Cat#ab69506 |

| | | |
|---|---|---|
| Anti-VEGFR2 | Immunoway | Cat#YT5845 |
| Anti-CD36 | Immunoway | Cat#YT5585 |
| Anti-Galectin-1 | GeneTex | Cat#GTX10156 |
| Anti-flag | Sigma | Cat#1804 |
| Anti-ANGPTL3 | Proteintech | Cat#11964-1-AP |
| Anti-ANGPTL4 | Proteintech | Cat#18374-1-AP |
| Anti-UCP1 | Abcam | Cat#ab10983 |
| **Chemicals, peptides, and recombinant proteins** | | |
| 3-isobutyl-1-methylxanthine (IBMX) | Sigma | Cat# I-5879 |
| Insulin | Sigma | Cat#91077C |
| Dexamethasone | Sigma | Cat# D-1756 |
| H-89 | Sigma | Cat# B1427 |
| Lipofectamine 2000 | Invitrogen | Cat# 11668019 |
| Anti-Flag M2 agarose beads | Sigma | Cat# F2426 |
| 3× Flag peptide | Sigma | Cat# F4799 |
| Mouse galectin-1 | Sino Biological | Cat#50100-MNAE |
| AA1 | Home-made[41] | N/A |
| AA98 | Home-made[40-41] | N/A |
| CD146 | Home-made | N/A |
| ANGPTL2 | Home-made | N/A |
| **Critical commercial assays** | | |
| QIAquick PCR purification kit | Qiagen | Cat#28106 |
| Triglyceride determination kit | Applygen | Cat#E1013 |
| Enhanced chemiluminescence assay kit | Pierce | Cat#34076 |
| Dual-luciferase reporter assay system | Promega | Cat# E1910 |
| Chromatin immunoprecipitation (ChIP) assay kit | Millipore | Cat#17-295 |

| | | |
|---|---|---|
| cAMP direct immunoassay kit (colorimetric) | Biovision | Cat#K371 |
| Mouse adiponectin ELISA kit | Proteintech | Cat# KE10044 |

**Table S3. Clinical sample information**

| Gender | Age | Weight (kg) | Height (m) | BMI | Location |
|---|---|---|---|---|---|
| Male | 49 | 52 | 1.70 | 18.0 | omentum fat |
| Male | 57 | 55 | 1.68 | 19.5 | omentum fat |
| Male | 51 | 59 | 1.70 | 20.4 | omentum fat |
| Male | 63 | 57 | 1.67 | 20.4 | omentum fat |
| Female | 58 | 55 | 1.61 | 21.2 | omentum fat |
| Female | 71 | 51 | 1.55 | 21.2 | omentum fat |
| Female | 74 | 61 | 1.63 | 23.0 | omentum fat |
| Male | 32 | 72 | 1.75 | 23.5 | omentum fat |
| Male | 53 | 78 | 1.76 | 25.2 | omentum fat |
| Male | 85 | 70 | 1.65 | 25.7 | omentum fat |
| Female | 84 | 64 | 1.52 | 27.7 | omentum fat |
| Male | 47 | 75 | 1.63 | 28.2 | omentum fat |
| Male | 55 | 87 | 1.70 | 30.1 | omentum fat |
| Female | 74 | 61 | 1.63 | 23.0 | omentum fat |
| Female | 38 | 55 | 1.58 | 22.0 | omentum fat |
| Male | 54 | 67 | 1.68 | 23.7 | omentum fat |
| Male | 76 | 70 | 1.64 | 26.0 | omentum fat |
| Male | 46 | 88 | 1.80 | 27.2 | omentum fat |
| Male | 65 | 80 | 1.68 | 28.3 | omentum fat |

| | | | | | |
|---|---|---|---|---|---|
| Female | 42 | 75 | 1.56 | 30.8 | omentum fat |
| Female | 73 | 63 | 1.62 | 24.0 | omentum fat |
| Female | 52 | 69 | 1.58 | 27.6 | omentum fat |
| Male | 65 | 73 | 1.72 | 24.7 | omentum fat |
| Male | 48 | 65 | 1.72 | 22.0 | omentum fat |
| Male | 59 | 91 | 1.72 | 30.8 | omentum fat |
| Male | 63 | 65 | 1.72 | 22.0 | omentum fat |
| Male | 78 | 75 | 1.69 | 26.3 | omentum fat |
| Male | 49 | 52 | 1.58 | 20.8 | omentum fat |
| Female | 58 | 60 | 1.64 | 22.3 | omentum fat |
| Female | 62 | 50 | 1.56 | 20.5 | omentum fat |
| Female | 49 | 70 | 1.61 | 27.0 | omentum fat |
| Male | 42 | 70 | 1.71 | 23.9 | omentum fat |
| Female | 35 | 62 | 1.60 | 24.2 | omentum fat |
| Male | 67 | 90 | 1.72 | 30.4 | omentum fat |
| Female | 42 | 69 | 1.60 | 27.0 | omentum fat |
| Male | 58 | 79 | 1.76 | 25.5 | omentum fat |

**Table S4. RT-qPCR primer sequences**

| Primer | Sequence |
|---|---|
| *mβ-actin F* | 5’- TCCTGTGGCATCCATGAAACT -3’ |
| *mβ-actin R* | 5’- TGGTACCACCAGACAGCACTGT -3’ |
| *mCd146 F* | 5’-AGAGCTCAGGGAGGTTGCTA -3’ |
| *mCd146 R* | 5’-ACTAGGCGTGCACTCAGAAC -3’ |
| *mF4/80 F* | 5’- AGGGTATCATGAGTTGATGGCA -3’ |
| *mF4/80 R* | 5’- TGTACCGTTGAAATAGGACGTG -3’ |
| *mMcp-1 F* | 5’- GACCCCAAGAAGGAATGGGT-3’ |
| *mMcp-1 R* | 5’- ACAGAAGTGCTTGAGGTGGTT-3’ |
| *mIL-6 F* | 5’-TGATGGATGCTACCAAACTGGA -3’ |
| *mIL-6 R* | 5’- TGTGACTCCAGCTTATCTCTTGG -3’ |
| *mIL-1β F* | 5’- TGCCACCTTTTGACAGTGATG -3’ |
| *mIL-1β R* | 5’- AAGGTCCACGGGAAAGACAC -3’ |
| *mTnf-α F* | 5’- GTAGCCCACGTCGTAGCAA -3’ |
| *mTnf-α R* | 5’- TAGCAAATCGGCTGACGGTG -3’ |
| *mCcl2 F* | 5’- AGATGCAGTTAACGCCCCAC-3’ |
| *mCcl2 R* | 5’- CCCATTCCTTCTTGGGGTCA -3’ |
| *mPparγ F* | 5’- GCCGAGGACACGAGGAAA -3’ |
| *mPparγ R* | 5’- CCGTTTTGGAATTGAGTGACTGA -3’ |
| *mC/ebpα F* | 5’- TTCGGGTCGCTGGATCTCTA -3’ |
| *mC/ebpα R* | 5’- GCGGAAAGTCTCTCGGTCTC -3’ |
| *mFabp4 F* | 5’-GGATTTGGTCACCATCCGGT -3’ |
| *mFabp4 R* | 5’- CCAGCTTGTCACCATCTCGT-3’ |
| *mPref-1 F* | 5’- CCCGGCCATCTGCTTCAC -3’ |
| *mPref-1 R* | 5’- TGATATTGACCGCCAGCTCC -3’ |
| *mCd36 F* | 5’- TCAACAGTCTCCCTGAAGCCA -3’ |
| *mCd36 R* | 5’- CACACATTTCAGAAGGCAGCAAC -3’ |
| *mGlut4 F* | 5’- CGGATAGGGAGCAGAAACCC -3’ |

| | |
|---|---|
| *mGlut4 R* | 5’- GAATCTAGAGGCCTCGGTGC -3’ |
| *mFasn F* | 5’- TGGAGAGCCCCACACACA -3’ |
| *mFasn R* | 5’- GCTGCGGAAACTTCAGGAAAT -3’ |
| *mAcc F* | 5’- ACAAAGTTGCTCTGAAAACAAATCA -3’ |
| *mAcc R* | 5’- TGACAGACTGATCGCAGAGAAAG -3’ |
| *mDgat1 F* | 5’- TGTGGTTAACCTGGCCACAA -3’ |
| *mDgat1 R* | 5’- AAACACGGAACCCACTGGAG -3’ |
| *mDgat2 F* | 5’- AAGAAAGGTGGCAGGAGATCG -3’ |
| *mDgat2 R* | 5’- GCAGGTTGTGTGTCTTCACC -3’ |
| *mCebpβ F* | 5’- CAAGCTGAGCGACGAGTACA -3’ |
| *mCebpβ R* | 5’- AGCTGCTCCACCTTCTTCTG -3’ |
| *mUcp-1 F* | 5’- CGTACCAAGCTGTGCGATGT -3’ |
| *mUcp-1 R* | 5’- GAAGCCACAAACCCTTTGAAAA -3’ |
| *mUcp-2 F* | 5’- AAGTGTTTCGTCTCCCAGCC -3’ |
| *mUcp-2 R* | 5’- GGAGTTCTGGAGGCTGCTTT-3’ |
| *mPpargc1□ F* | 5’- GTCATGTGACTGGGGACTGT -3’ |
| *mPpargc1□ R* | 5’- CAACCAGAGCAGCACACTCT -3’ |
| *mPpargc1□ F* | 5’- TCCTGTAAAAGCCCGGAGTAT -3’ |
| *mPpargc1□ R* | 5’- GCTCTGGTAGGGGCAGTGA -3’ |
| *mPrdm16 F* | 5’- CCACCAGCGAGGACTTCAC -3’ |
| *mPrdm16 R* | 5’- GGAGGACTCTCGTAGCTCGAA -3’ |
| *mAdrb3 F* | 5’- GGCCCTCTCTAGTTCCCAG -3’ |
| *mAdrb3 R* | 5’- TAGCCATCAAACCTGTTGAGC -3’ |
| *mDio2 F* | 5’- GAAGGCTGCCGAATGTCAAC -3’ |
| *mDio2 R* | 5’- GCGTGAGCAGTCTTGTTTGG -3’ |
| *mCidea F* | 5’- TGACATTCATGGGATTGCAGAC -3’ |
| *mCidea R* | 5’- GGCCAGTTGTGATGACTAAGAC -3’ |
| *mPpar□ F* | 5’- AGAGCCCCATCTGTCCTCTC -3’ |
| *mPpar□ R* | 5’- ACTGGTAGTCTGCAAAACCAAA -3’ |
| *mCpt1□ F* | 5’- GCACACCAGGCAGTAGCTTT -3’ |

| | |
|---|---|
| *mCpt1□ R* | 5’- CAGGAGTTGATTCCAGACAGGTA -3’ |
| *mMcad F* | 5’- AGGGTTTAGTTTTGAGTTGACGG -3’ |
| *mMcad R* | 5’- CCCCGCTTTTGTCATATTCCG -3’ |
| *mLcad F* | 5’- TCTTTTCCTCGGAGCATGACA -3’ |
| *mLcad R* | 5’- GACCTCTCTACTCACTTCTCCAG -3’ |
| *mAdiponectin F* | 5’- TACTGCAACATTCCGGGACTC -3’ |
| *mAdiponectin R* | 5’- TTGGTCTCCCACCTCCAGAT -3’ |
| *mPDGFR□ F* | 5’- ACTCCATGGGTGGAGATTCG -3’ |
| *mPDGFR□ R* | 5’- CTCATAGCGTGGCTTCTTCTGC-3’ |
| *mVEGFR2 F* | 5’- GCCTCTGTGGGTTTGCCT -3’ |
| *mVEGFR2 R* | 5’- GAATCACGCTGAGCATTGGG -3’ |
| *hCD146 F* | 5’- GGAGCCAAACATCCAGGTCA -3’ |
| *hCD146 R* | 5’- ACCACTCGACTCCACAGTCT -3’ |
| *hβ-actin F* | 5’-CCTCGCCTTTGCCGATCC -3’ |
| *hβ-actin R* | 5’- AGGAATCCTTCTGACCCATGC-3’ |
| *hFASN F* | 5’- AGATGGCTTGCTGGAGAACC -3’ |
| *hFASN R* | 5’- AAGCCGTAGTTGCTCTGTCC -3’ |
| *hDGAT1 F* | 5’- CCAGTGACCTCTTCAGCCTGC -3’ |
| *hDGAT1 R* | 5’- TTTCTAGGTAGGGGAGTGTGGG -3’ |
| *hDGAT2 F* | 5’- GGTCTGGGAGATGGGGAGTG -3’ |
| *hDGAT2 R* | 5’- ACCTCCTGCCACCTTTCTTG -3’ |
| *hACC F* | 5’- GGAACTGCGGCTTGTGTTTA -3’ |
| *hACC R* | 5’- GACATGCTGGACCTTTGAAGCA -3’ |
| *hSCD1 F* | 5’- CTGGCTTGCTGATGATGTGC -3’ |
| *hSCD1 R* | 5’- CCGGGGGCTAATGTTCTTGT -3’ |
| *mCpt1□ F* | 5’- GGACAGGAGTGAACCCGAG -3’ |
| *mCpt1□ R* | 5’- ATGCGGATCAGGCGTTTCTT -3’ |
| *mMcad F* | 5’- TGCTGCAGGAGTTCACCGAA -3’ |
| *mMcad R* | 5’- CCAAGTCCAAGACCTCCACA -3’ |
| *mLcad F* | 5’- TGGAGGGGATCTGTACTCCG -3’ |

| | |
|---|---|
| *mLcad R* | 5’- AGCTCCAGGCTCTGTCATTG -3’ |
